# The Impact of Adverse Childhood Experiences on Cognitive Control Across the Lifespan: A Systematic Review and Meta-analysis of Prospective Studies

**DOI:** 10.1177/15248380241286812

**Published:** 2024-10-13

**Authors:** Satwika Rahapsari, Liat Levita

**Affiliations:** 1 School of Psychology, University of Sheffield, UK; 2 Faculty of Psychology, Universitas Gadjah Mada, Indonesia; 3 School of Psychology, University of Sussex, UK

**Keywords:** adverse childhood experiences, ACEs, cognitive control, working memory, cognitive flexibility, inhibitory control, meta-analysis

## Abstract

Adverse childhood experiences (ACEs) are strongly associated with impaired cognitive control, yet research on ACEs’ effects across cognitive control domains—working memory, cognitive flexibility, and inhibitory control—remains sparse. This systematic review and meta-analysis evaluated the overall impact of ACEs on each of these cognitive control domains and explored moderating factors such as age, gender, cognitive control paradigms, and ACEs subtypes based on the dimensional model of adversity and psychopathology. A database search was conducted in SCOPUS, MedLine, PsycINFO, and Web of Science. Only prospective studies were included to ensure temporal order inferences, with at least two data collection points, assessing ACEs at baseline (T1) and cognitive control during follow-up (T2). Thirty-two studies (*N* = 26,863) producing 124 effect sizes were analyzed. Three-level meta-analyses revealed small-to-medium negative associations between ACEs and overall cognitive control (*g* = −0.32), and in each domain: working memory (*g* = −0.28), cognitive flexibility (*g* = −0.28), and inhibitory control (*g* = −0.32). The negative associations between ACEs and cognitive control were consistent across age, gender, and cognitive control paradigms. ACEs subtypes moderated the association with cognitive flexibility (*p* = .04) but not working memory or inhibitory control. Specifically, the deprivation subtype exhibited a stronger negative association with cognitive flexibility compared to threat and threat-and-deprivation subtypes. These findings highlight the pervasive negative impact of ACEs on cognitive control across ages and emphasize the need for targeted interventions. Implications, current gaps, limitations in research, and future study recommendations are discussed.

## Introduction

Adverse childhood experiences (ACEs) comprise a broad spectrum of exposure to adverse events during childhood. These experiences include maltreatment (e.g., emotional, physical, sexual abuse, and neglect), household dysfunctions (e.g., financial difficulty, domestic abuse, and parental separation), and other negative experiences like exposure to warfare or conflict and community violence (Felitti et al., 1998; Javier et al., 2019). The detrimental effects of ACEs on individuals are well-documented. ACEs are linked to significant behavioral and mental health issues, including suicidal tendencies, depression, internalizing disorders, and anxiety (see Sahle et al., 2022 for review). Furthermore, individuals with ACEs are at an increased risk of developing physical health issues, such as obesity, diabetes, heavy alcohol use, cancer, and heart disease (Hughes et al., 2017). Over recent years, studies have also begun to investigate the ACEs’ impact on individuals’ neurocognitive functioning (McCrory et al., 2017; Pollok et al., 2022). One neurocognitive function that has been repeatedly reported to be negatively associated with ACEs is cognitive control (e.g. Danese et al., 2017; Motsan et al., 2022; Mueller et al., 2010; Wade et al., 2020), both in clinical and healthy populations (Marshall et al., 2016).

Cognitive control encompasses a collection of higher-level cognitive processes that allow information processing, responding, and adapting to perform goal-relevant behaviors (Diamond, 2013; Gratton et al., 2018). The majority of neuropsychological studies suggest three primary cognitive control domains—working memory, cognitive flexibility, and inhibitory control (Diamond, 2013; Miyake et al., 2000). Working memory refers to the cognitive potential to integrate novel information, monitor, and remove irrelevant information (Goldstein et al., 2014). Inhibitory control involves the capacity to regulate attention, emotions, and mental processes, leading to the suppression of prevailing, automatic, or instinctive reactions (Diamond, 2013; Miyake et al., 2000). The last is cognitive flexibility, or the capacity to effortlessly transition between different tasks or goals (Diamond, 2013). Developing proficiency in these cognitive skills is essential not only for meeting short-term objectives but also has been associated with improved academic achievement (Samuels et al., 2016) and the abilities to regulate emotion (Hendricks & Buchanan, 2016).

### The Associations Between ACEs and Cognitive Control

Stress and adversity can result in the sustained release of the stress hormone cortisol, which can disrupt the control of the hypothalamic-pituitary-adrenal (HPA) axis (Kalmakis et al., 2015; Miller et al., 2007). This prolonged activation heightens vigilance during stress but can have long-term negative effects, reducing stress tolerance and intensifying stress responses (Tarullo & Gunnar, 2006). HPA axis dysregulation also impacts brain development, particularly in key regions critical for cognitive control such as the prefrontal cortex (PFC). The PFC, which is essential for cognitive control processes such as strategic planning and decision-making (Diamond, 2002), undergoes significant development during childhood and adolescence (Gogtay et al., 2004; Larsen & Luna, 2018; Luna et al., 2015) and shows vulnerability to chronic stress associated with ACEs (McEwen & Morrison, 2013). Indeed, stress-induced rise in allostatic load adversely affects the PFC’s structure and functions (Herzberg & Gunnar, 2020; Lupien et al., 2015), and a recent meta-analysis in adults has shown that elevated allostatic load correlates with decreased cognitive control abilities (D’Amico et al., 2020).

Consistent with this, studies have reported the relationships between ACEs and impairments in inhibitory control (e.g., Demers et al., 2022; Fava et al., 2019; Schäfer et al., 2023), as well as poorer cognitive flexibility (Harms et al., 2018; Kalia et al., 2021). Additionally, Goodman et al. (2019), in their systematic review and meta-analysis of 23 studies, showed that ACEs are associated with poorer working memory in adults.

However, while these studies have found a link between ACEs and cognitive control, to date, there is no comprehensive review and no report on the total effect sizes of ACEs’ impact on cognitive control and each cognitive control domain, and whether this impact manifests itself distinctly at different age. Obtaining this information is crucial because it will provide a clearer understanding of the magnitude and nuances of ACEs’ effects, enabling more targeted and effective interventions. For instance, by identifying which subtype of ACEs are most linked to a specific cognitive control domain, a more tailored intervention could be developed, aimed at strengthening that particular cognitive control domain in individuals with ACEs. Moreover, understanding the impact across the lifespan can inform age-specific strategies to support individuals affected by ACEs. This knowledge is vital for policymakers, educators, and healthcare providers to allocate resources efficiently and develop comprehensive prevention and treatment programs.

Prior to conducting this study, no existing systematic reviews and meta-analyses that have examined the impact of ACEs on cognitive control in all ages were found after an initial search on SCOPUS and Web of Science, as well as PROSPERO (International Prospective Register of Systematic Reviews) pre-registration.

Nevertheless, previous research has laid a foundational understanding, such as a systematic review of 36 studies that summarized the detrimental effects of ACEs on cognitive control in children (Lund et al., 2020). Lund et al. (2020) reported that various forms of ACEs, including exposure to intimate partner violence, neglect, abuse, and maternal depression, were associated with poor cognitive control outcomes. Likewise, another systematic review of prospective cohort studies reported that ACEs, especially neglect, were significantly associated with lower cognitive control, even when adjusted for sociodemographic variables (Su et al., 2019). However, while providing a comprehensive review of the associations between ACEs and cognitive control, these two reviews did not quantitatively meta-analyze the effect sizes; thus, the overall magnitude of the association between ACEs and each cognitive control domain could not be observed. Therefore, a meta-analysis study is needed to quantitatively assess the total effect size of the associations between ACEs and cognitive control domains, as this will provide a clearer understanding of the magnitude and variability of these relationships. This is important because having precise estimates of the effect sizes enables a more accurate assessment of how strong the association between ACEs and cognitive control is. Moreover, understanding the variability in these relationships can help identify which subtype of ACEs has the biggest impact on cognitive control, which is essential for developing targeted and effective interventions.

Previous meta-analyses have enhanced our understanding of the relationships between ACEs and cognitive control, but they have some limitations that this current study aims to address. First, the past meta-analysis studies conducted by Johnson et al. (2021) and Op Den Kelder et al. (2018) have reported the negative impact of ACEs on each cognitive control domain—working memory, inhibitory control, and cognitive flexibility. However, these studies have only focused on children and adolescents and did not incorporate research on the adult population for their meta-analyses, thereby neglecting the potential long-term impact of ACEs in adulthood. This narrow age range leaves a critical gap in understanding how ACEs affect cognitive control across the lifespan. Since neurocognitive functions show prolonged maturational development, it is imperative to determine whether ACEs also impact adults’ cognitive control.

Further, both past studies (Johnson et al., 2021; Op Den Kelder et al., 2018) combined the results from cross-sectional and longitudinal research in their meta-analyses. While this is a common practice to obtain a broad picture of the overall effect size and reveal more insights into the relationship between ACEs and cognitive control, it may limit the ability to infer the chronological sequence between ACEs and cognitive control. Retrospective research is susceptible to recollection bias due to the potential for erroneous assessment of ACEs, which makes them a less-than-ideal method for researching relationships that depend on time (Hänninen & Soininen, 1997; Hardt & Rutter, 2004; Reuben et al., 2016). This limitation could hinder our current understanding of how ACEs affect cognitive control prospectively.

Comparing cross-sectional and longitudinal research may offer additional insights into the relationship between ACEs and cognitive control. However, it is critical to acknowledge that the results may be influenced by the large number of cross-sectional studies incorporated into the meta-analyses, which could introduce bias. For instance, the previous review (Johnson et al., 2021) compared the effect sizes from longitudinal and cross-sectional studies and found no significant difference in effect sizes resulting from both study designs. Nevertheless, the result could be biased because their analysis included a limited number of longitudinal studies (only 20 out of 91 studies) that were included in their analysis. Given the limited number of longitudinal research conducted, focusing exclusively on prospective or longitudinal studies can provide a more robust and reliable estimate of the associations between ACEs and cognitive control. Mixing cross-sectional and longitudinal data can introduce heterogeneity, complicating the interpretation of results and potentially obscuring true relationships (Higgins et al., 2023). By concentrating on prospective studies, we may achieve a clearer, more comprehensive knowledge of the lasting impacts of ACEs on cognitive control development.

In addition, some studies in the existing meta-analyses used self-report or teacher/caregiver reports to measure cognitive control, which can infer biased results compared to performance-based cognitive control measurement. Research has reported a weak relationship between self-report and behavioral cognitive control measurements (Saunders et al., 2018), indicating that these two types of measurements, which are assumed to represent the same construct, are not strongly associated with each other. This tenuous correlation implies that self-report and behavioral measures may have underlying differences and hence cannot be regarded as interchangeable markers of the same construct (Dang et al., 2020).

### Current Study

Consequently, this current study included studies across the lifespan to examine the potential moderating effects of age on the impact of ACEs on cognitive control. In addition, this study only incorporated prospective studies to reduce the uncertainty concerning temporal biases affecting observed outcomes. Furthermore, for enhanced objectivity and domain-specific assessment, only studies employing cognitive control behavioral tasks were included.

Other than age, several key factors that might also affect the association between ACEs and cognitive control were also investigated through moderation analysis. These factors, which encompassed study type (correlational and comparative), biological sex differences, and the specific cognitive control task used, were investigated to understand how they might influence the association between ACEs, and each analyzed cognitive control domain.

First, considering study type as a moderator is important because this could be a source of heterogeneity or the variation of results from one study to another since the nature of effect size reported in correlational versus comparative studies is different. Correlational studies usually report effect sizes as correlation coefficients, which show both the strength and direction of relationships between variables. In contrast, effect sizes in comparative studies might be reported as mean differences, reflecting the magnitude of differences between groups of conditions (Kettler, 2019). For example, a correlational study investigating the association between ACEs and cognitive flexibility (e.g., Lewis-Morrarty et al., 2012) showed a slightly larger effect size (*g* = −0.10) compared to similar research using a comparative study design (*g* = −1.10) (e.g., Savopoulos et al., 2022). Even though the effect sizes of all studies were converted in Hedges’ *g* for this current meta-analysis, these different metrics may lead to variability in the magnitude and interpretation of effect size, necessitating their consideration as a moderating factor.

Additionally, it is essential to consider biological sex as a moderator for conducting moderator analysis. This approach will enable a comprehensive knowledge of how sex differences affect the association between ACEs on cognitive control. Studies have demonstrated that men and women exhibit different patterns of brain development (Beck et al., 2023), notably in areas linked to cognitive control, such as the PFC, and that sex steroid hormones interact with key neurotransmitters involved in cognitive control, such as GABA, dopamine, serotonin, and glutamate (Barth et al., 2015; Halari et al., 2006). Furthermore, there are differences between men and women in the timing of puberty, with girls typically experiencing puberty earlier than boys (Juraska & Willing, 2017). The pubertal timing differences are linked to the later maturation of PFC (Blakemore, 2008; Herting et al., 2015). It is suggested that the varying paths of regional brain maturation may partially account for the disparities in cognitive control development between sexes (Rubia et al., 2013; Spielberg et al., 2015). In addition, it is worth noting that men and women display distinct physiological reactions to stress, which are driven by hormonal and brain maturation differences (Bale & Epperson, 2015, for review). Taken together, these biological differences may result in the sex-specific impact of ACEs on cognitive control.

Further, cognitive control tasks may vary significantly in their design and the specific cognitive demands they place on participants. For instance, although all are inhibitory control tasks, tasks like the Go/No-go Task and Stroop Test, each measure different aspects of inhibitory control, such as response inhibition and interference control, respectively (Friedman & Miyake, 2004; Nigg, 2000). In addition, the complexity of tasks also can differ, impacting the cognitive load and the strategies participants use to complete them. Tasks that are more complex or require multi-step problem-solving may show different effects compared to simpler tasks (Miyake et al., 2000; Spedden et al., 2017). Therefore, considering the task paradigm as a moderator may help explain heterogeneity in effect sizes across studies. Different tasks might produce different magnitudes of effects.

Notably, this study also examined how different types of ACEs might influence the outcomes and applied the Dimensional Model of Adversity and Psychopathology (DMAP; McLaughlin & Sheridan, 2016) to cluster the ACEs subtypes in the studies analyzed in the meta-analysis. DMAP suggests that ACEs encompass core dimensions that are closely linked to the negative effect of ACEs on psychopathology as well as neurocognitive functions. Those core ACEs dimensions are classified into threat and deprivation subtypes. The threat subtype refers to situations where there exists real danger or intimidation to the child’s physical well-being, for example, exposure to violence or abuse in family or community settings. On the other hand, deprivation refers to situations characterized by the lack of environmental stimuli such as neglect (physical and/or emotional), growing up in an institutional setting, and/or facing food insecurity. According to DMAP, the absence of environmental complexity and stimulation, which is a defining feature of deprivation, will have a greater impact on cognitive control compared to exposure to threat. This is due to the hypothesized link between deprivation and the brain networks responsible for cognitive control (McLaughlin & Sheridan, 2016; McLaughlin, Sheridan, Winter, et al., 2014).

Previous research has indicated that specific dimensions or subtypes of ACEs could also influence how ACEs are linked to cognitive control. However, results from studies investigating moderator analysis in this regard have thus far been inconclusive. For instance, Johnson et al. (2021) found that the deprivation subtype of ACEs is more likely to have a greater impact on working memory and inhibitory control but not cognitive flexibility. In contrast, Op Den Kelder et al. (2018) found that the association between ACEs and cognitive flexibility is stronger in cases of exposure to deprivation. Consequently, there is a need for a meta-analysis study incorporating the latest research to validate and reconcile these disparate findings.

Drawing from previous theories and research, it was predicted that ACEs would be significantly and negatively associated with each domain of cognitive control. As the previous research was inconclusive about how variables such as the subtypes of ACEs and cognitive control measurement task moderated the associations between ACEs and cognitive control, we did not develop a hypothesis regarding possible moderating effects and instead explored them through moderator analysis. The results of this systematic review and meta-analysis could offer insights for future research direction on the topic of ACEs and their impact on cognitive control. In addition, the findings may offer insights that could potentially inform targeted prevention and intervention strategies for individuals exposed to ACEs. For example, by understanding the specific subtypes of ACEs that are most strongly associated with a particular cognitive control domain, more tailored interventions could be developed to bolster this specific cognitive control domain in individuals experiencing high risk of ACEs exposure.

## Methods

This systematic review was designed and conducted, and subsequent findings were presented following the guidelines set out in the Preferred Reporting Items for Systematic Reviews and Meta-Analyses (PRISMA) 2020 statement (Page et al., 2021). The study protocol detailing the research question, criteria for inclusion, search approach, and methods of analysis was pre-registered with PROSPERO CRD42021298454 (Rahapsari et al., n.d.).

### Literature Search Method

A systematic database search for studies was conducted through the electronic databases of Scopus, PsycINFO, MedLINE, and Web of Science (Core Collection). The search was initially carried out on December 2, 2022, and an update search was conducted on December 27, 2023.

Strings using Boolean search terms were planned thematically based on ACEs’ exposure, cognitive control outcome, and study design. Each thematic string was joined with OR, and distinct strings were combined with AND. When conducting searches in the Ovid database, these strings were converted into Medical Subject Heading (MeSH) terms (see Supplemental Information 1 for detailed search strategies).

**Figure 1.**
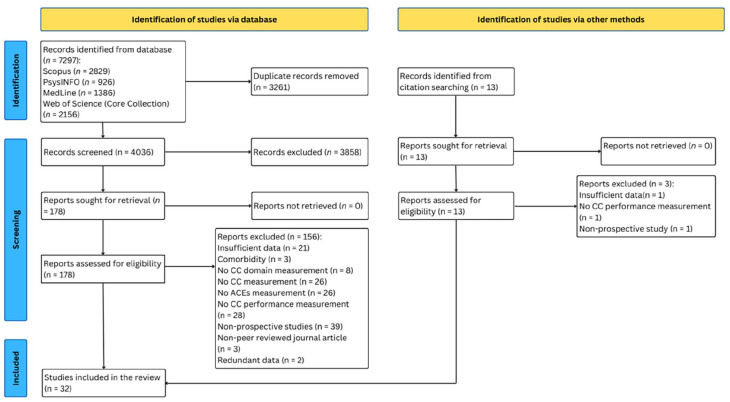
PRISMA flowchart of systematic search and study selection. *Source.* Adapted from Page et al. (2021). *Note*. PRISMA = Preferred Reporting Items for Systematic Reviews and Meta-Analyses.

Figure 1 demonstrates the process of searching and selecting the articles. The search has resulted in 7,297 references. Subsequently, the bibliographies of relevant research were examined to uncover eligible studies that might not have been captured in the database searches, which yielded 13 additional articles. After removing duplicates and conducting initial screening based on titles and abstracts, 178 full-text articles were evaluated to determine their eligibility for inclusion.

### Inclusion and Exclusion Criteria

After removing the duplicates, titles, and abstract screening for initial eligibility, the screening was conducted. Further work was full-text screening for potentially eligible articles and assessing for inclusion.

To ascertain whether a primary study was suitable for inclusion in this review, inclusion criteria were determined: (a) The study must include human participants of all ages; animal studies were eliminated. (b) The study design utilized quantitative measurement; therefore, systematic reviews and qualitative studies were removed. (c) The study needed to be an original research article published in a peer-reviewed journal; books, dissertations, and editorials were excluded. While including dissertations in the study may mitigate publication bias by encompassing studies regardless of their outcomes, this meta-analysis opted to exclude dissertations for several reasons. First, unlike peer-reviewed journal articles, dissertations typically undergo minimal scrutiny and may lack the rigorous peer review and editorial oversight that journals provide. Exclusion ensures a more balanced and representative synthesis of the available evidence. Additionally, dissertations are often not easily accessible or widely available to researchers, particularly those outside academic institutions. By excluding dissertations, the review aims to ensure that its findings are based on a comprehensive and accessible body of literature. (d) The study had to use longitudinal or prospective design, which had a clear temporal order between ACEs and cognitive control; the study also required data collection at a minimum of two time points, with measurement of ACEs (at T1) preceding assessment of the cognitive control outcomes (at T2); thus, cross-sectional studies were excluded. (e) The study needed to employ validated batteries of neuropsychological measurements, or measurements that were carefully designed and rigorously tested sets of assessments that provide reliable, accurate, and comprehensive evaluations of cognitive control, for example, Cambridge Neuropsychological Test Automated Battery (CANTAB), Multi-Source Interference Task, and Dimensional Change Card Sort. These measurement tools should measure the performance indicators of at least one cognitive control domain (i.e., working memory, cognitive flexibility, inhibitory control). (f) The studies included in this systematic review and meta-analysis should measure one or more exposure to ACEs using well-accepted measurement tools, ensuring a comprehensive capture of childhood adversity. ACEs refer to a range of stressful or traumatic events occurring during childhood (<18 years), such as physical, sexual, and emotional abuse, neglect, financial crisis, and mental illness of caregivers. In this review, a well-accepted measurement of ACEs is defined as a method of measurement that has been validated and widely used in research and clinical settings to assess these experiences. Such measurements can include institutional records, parent reports, and self-reports. Institutional records may include documents of cases of abuse or neglect from social services or healthcare providers. Parent reports involve caregivers’ accounts of the child’s experiences, often collected through structured interviews or questionnaires. Self-reports are assessments completed by the individuals themselves typically using standardized questionnaires to capture ACEs. (g) The study needed to present a minimum of one effect size indicating the relationship between ACEs and cognitive control or provide adequate statistical data to compute at least one effect size. Of the 178 full-text articles assessed, 32 studies were eligible, yielding *k* = 124 effect sizes. The list of references of the included studies can be found in Supplemental Information 2.

### Data Extraction

The next step is to extract data from the selected articles. Based on a coding protocol outlining the required information and extraction methods, two coders (S.R. and L.L.) extracted pertinent data from each study. The coding protocol and the study data can be seen on the authors’ Open Science Framework (OSF) page (https://osf.io/4g8af/). Independently, two coders inputted and archived their data within an internal lab folder. Subsequently, they jointly piloted and refined the protocol using five studies. Any disparities in coding were addressed collaboratively by the coders.

The following criteria were used to code the extracted information in each study: (a) author(s) of the study, (b) publication year, (c) study type (correlation, comparison), (d) country of data collection, (e) sample size, (f) sex (percentage of females), (g) mean and standard deviation of age, (h) cognitive control domain (working memory, cognitive flexibility, inhibitory control), (i) cognitive control measurement tool(s), (j) measurement tool(s) of ACEs, (k) ACEs subtypes (threat, deprivation, threat, and deprivation), (l) effect size (i.e., mean and standard deviation for comparative studies, zero-order correlation coefficient for correlational studies, or effect size approximations (e.g., *t* statistics) if means and standard deviations or correlation coefficient were not provided). Effect sizes were not adjusted; results were not controlled for sex, socioeconomic status (SES), education level, and/or age.

### Effect Size Calculation

To calculate the effect sizes, for studies reporting effects for both a full sample and a subsample, only the data from the full sample was coded. For instance, if a study had 100 participants but only 95 completed the measurement, data was coded for the 95 participants who provided complete responses. This approach ensures that the analysis is based on the most comprehensive and accurate data available from each study.

To quantitatively synthesize the findings across studies, Hedges’ *g* was employed to calculate the effect sizes. Hedges’ *g* was chosen as it provides a standardized measure of association that adjusts for biases caused by small sample sizes, ensuring a more accurate estimation of effect magnitude (Durlak, 2009; Hedges, 1981).

The effect sizes were computed based on provided means and standard deviations. In cases where means and standard deviations were not available, Hedges’ *g* was converted from correlation coefficient or approximations of effect size (Borenstein et al., 2021). For each study included in the meta-analysis, means and standard deviations for the groups being compared. When these statistics were reported, Hedges’ *g* was directly calculated using the formula:

$${\mathrm{Hedges}}^{'}g=\frac{M_{1}-M_{2}}{SD_{pooled}}$$

where *M*_1_ and *M*_2_ are the means of the two groups, and *SD*_pooled_ is the pooled standard deviation.

In cases where means and standards were not provided, Hedges’ *g* was derived from other available statistical data. Specifically, when correlation coefficients (*r*) were available, *r* transformed into Hedges’ *g*. Further, for studies that provided other effect size metrics, such as *t*-values, these metrics were converted into Hedges’ *g* using established conversion formulas. The conversion of effect size metrics into Hedges’ *g* was conducted with the esc package (Lüdecke, 2018) using the 4.2.3 version of R (R Core Team, 2022).

### Assessment of Risk of Bias and Study Quality

A tool of risk-of-bias assessment was applied to evaluate all the included studies. The risk of bias in eligible studies that are group comparison studies, such as comparisons between ACEs-exposed and non-ACEs-exposed and eligible non-experimental studies reporting correlations between ACEs exposure and cognitive control was assessed with an adaptation of the Newcastle–Ottawa Scale (NOS) assessment form for longitudinal studies (Wells et al., 2000). This analysis addresses the methodological limitations of studies, especially in measuring the cognitive control variable. The assessment comprised three domains to be assessed from the included studies: (a) selection, (b) comparability, and (c) outcome.

Study quality was categorized as poor, fair, and good based on the overall score. A study was categorized as poor if the selection domain scored 0 or 1, the comparability domain scored 0, or the outcome domain scored 0 to 1. Next, a study was categorized as fair if the selection domain scored 2, the comparability domain scored 1 or 2, and the outcome domain scored 2 or 3 points. Further, a study was categorized as good if the selection domain scored 3 or 4, the comparability domain scored 1 or 2, and the outcome domain scored 2 or 3. The maximum achievable quality score was 9. Any discrepancies in study quality coding (3 out of 32 articles) were resolved through discussions between two coders (S.R. and L.L.). Interrater reliability was *k* = 0.79. See Supplemental Information 5 for the complete result of the study quality assessment.

Further, to assess the potential for bias, the quality categorization of the articles from the NOS assessment was examined to ascertain if it influences the relationships between ACEs and cognitive control outcomes.

### Statistical Analyses

The standardized effect size (Hedges’ *g*) from all studies was synthesized using a three-level random-effects model, implemented using restricted maximum likelihood estimation. Subsequently, the moderator analyses were also conducted using the three-level meta-analyses model. This model allows us to partition the total variance into within-study and between-study components, providing a more accurate and comprehensive analysis of the effect sizes (Assink & Wibbelink, 2016).

Given the hierarchical nature of the data, a three-level meta-analysis was conducted to model dependence structure within and between studies. This model analyzed three sources of variance to account for dependencies in effect sizes using three-level random effects. First, at Level 1, it analyzed the variability in the observed effect sizes due to sampling (within-study variability). At Level 2, the variation among effect sizes obtained from the same study was examined (within-study correlation). Lastly, at Level 3, it calculated the between studies’ variances (Assink & Wibbelink, 2016). By employing the three-level meta-analyses, it was possible to model the interdependence of effect sizes and retain all pertinent information from the included studies. As a result, the three-level meta-analyses allow for the attainment of maximum statistical power, making it a robust method in comparison to the more conventional meta-analysis techniques.

Three separate three-level meta-analyses were carried out to investigate the association between ACEs and three domains of cognitive control: ACEs and working memory, ACEs and cognitive flexibility, and ACEs and inhibitory control. Cohen’s (2016) guidelines were employed to interpret the overall associations, with a threshold of .10 indicates a small effect size, .30 indicates a medium effect size, and .50 indicates a large effect size.

Further, moderator analyses were performed. Significant moderators were grouped, and pooled estimates were presented for each group. Statistical significance was established at α = .05. Potential moderating factors include chronological age (as continuous variable), study type (as categorical variable: correlation, comparison), sex (continuous variable of the percentage of female participants), outcome measurement paradigms (as categorized variable, e.g., for working memory, measurement paradigm were categorized as Digit Span, CANTAB Spatial Working Memory, and N-back), and ACEs subtypes as categorical variable (threat, deprivation, threat, and deprivation), were analyzed. The categorization of ACEs subtypes is based on the DMAP (McLaughlin & Sheridan, 2016) approach, which identifies ACEs into two main subtypes: deprivation (e.g., neglect, institutional rearing) and threat (e.g., violence, abuse). Due to existing studies that measure uncategorized ACEs (i.e., measured ACEs in general or cumulatively, and cannot be distinctly separated as threat or deprivation), the “threat and deprivation” category was added to accommodate ACEs subtypes that do not clearly represent threat or deprivation subtypes. The complete information on the study's characteristics included in the meta-analysis can be found in the Supplemental Information 3.

Separate mixed-effect model analyses were performed for each of the three-level meta-analyses to examine bivariate moderators individually.

Before conducting moderator analyses, an assessment was made to determine the extent of variation in the extracted effect sizes or to ascertain the presence of heterogeneity in effect sizes. Differences in effect sizes indicate that variations in the strength of identified relationships are likely influenced by factors such as study characteristics and sample demographics, rather than being random occurrences. To elaborate further, assess whether notable variability is discernible at Level 2 (effect sizes variability within the same studies) and/or Level 3 (effect sizes variability derived from different studies). Subsequently, moderator analysis can be performed to investigate whether certain variables influence the overall association significantly.

The dmetar package in R was employed to visualize the heterogeneity in effect sizes within the same study and across different effect sizes within the same cohort. Subsequently, Cochran’s *Q* and *I*^2^ statistics were applied to evaluate the significance of this heterogeneity.

Results from studies with a small sample size may be prone to bias. Therefore, to assess publication bias, the Egger’s regression model was used (Egger et al., 1997). This method determines the effect sizes’ nonindependence (Rodgers & Pustejovsky, 2021). Further, to illustrate the precision of effect sizes and to investigate potential publication bias, funnel plots were generated.

The analysis in this study was carried out using the 4.2.3 version of R (R Core Team, 2022) with the metafor package (Viechtbauer, 2010), following the R syntax for three-level meta-analyses outlined in the tutorial by Harrer et al. (2021). The parameters of the model were calculated utilizing the restricted maximum likelihood approach (Viechtbauer, 2005), with statistical significance defined as a two-tailed *p*-value below .05. R scripts and data are available on the authors’ OSF page (https://osf.io/4g8af/).

## Results

### Literature Search and Study Characteristics

The selection process of articles is displayed in Figure 1. A total of 7,297 articles were identified via electronic searches across 4 databases, and 3,261 duplicates were detected and removed. Subsequently, 4,036 abstracts underwent screening, leading to the identification of 178 articles eligible for full-text examination. Out of these, 32 studies fulfilled the inclusion criteria and were included into the review, yielding *k* = 124 outcomes extracted from the studies, representing 26,863 individuals.

Table 1 lists the characteristics of the studies and the quality assessment of the included articles. The majority of the studies were carried out in the United States (25 studies, *k* = 92), followed by the United Kingdom (4 studies, *k* = 14), Israel (1 study, *k* = 1), Germany (1 study, *k* = 9), and Australia (1 study, *k* = 8). Correlational design was used in 17 studies, yielding *k* = 61 outcomes, and comparative design was used in 15 studies, yielding *k* = 63 outcomes.

**Table 1.** Characteristics of Included Studies.

Author	Country	Study Design	*n*	Age *M* (*SD*)	Sex	CC Domain	ACEs Subtypes	Neurobiological Measure
Almas et al. (2016)	United States	Comparison	105	12.24 (0.59)	NA	WM	Deprivation	NA
Awada et al. (2023)	United States	Correlation	4,898	9 (NA)	NA	WM	Threat, deprivation	NA
Bosquet Enlow et al. (2019)	United States	Correlation	53	3.84 (0.25)	45	WM, IC	Threat and deprivation	NA
Brieant et al. (2022)	United States	Correlation	167	18.87 (0.61)	47	IC	Threat and deprivation	fMRI
Clark et al. (2022)	United States	Correlation	70	12.25 (1.78)	44.29	CF, IC	Threat	NA
Colvert et al. (2008)	United Kingdom	Comparison	160	11 (NA)	55	IC	Deprivation	NA
Conradt et al. (2014)	United States	Correlation	860	11 (NA)	49	WM	Threat and deprivation	Cortisol reactivity
Demers et al. (2022)	United States	Comparison	72	30.18 (3.47)	48.6	IC	Threat and deprivation	fMRI
Demeusy et al. (2018)	United States	Comparison	89	2.16 (NA)	52.8	WM	Deprivation	NA
Frenkel et al. (2020)	United States	Comparison	128	5.18 (0.17)	53.12	IC	Deprivation	EEG-ERP
Golm et al. (2021)	United Kingdom	Comparison	89	25.3 (NA)	50	IC	Deprivation	NA
Gustafsson et al. (2015)	United States	Correlation	154	5 (NA)	49	WM, CF, IC	Threat	NA
Harms et al. (2017)	United States	Correlation	38	20.6 (NA)	46.15	IC	Threat and deprivation	fMRI
Hunter et al. (2022)	United States	Correlation	6,337	29.03 (1.81)	51.9	WM	Threat and deprivation	NA
Jankowski et al. (2017)	United States	Comparison	25	12.22 (1.44)	52	IC	Deprivation	MRI
Kavanaugh et al. (2023)	United States	Correlation	53	11 (0.7)	53	IC	Threat and deprivation	EEG-ERSP
Kokosi et al. (2021)	United Kingdom	Correlation	4,525	10 (NA)	49.21	WM	Deprivation	NA
Lamm et al. (2018)	United States	Comparison	144	12.65 (0.47)	48.61	IC	Deprivation	EEG-ERP
Lengua et al. (2022)	United States	Correlation	306	5 (NA); 8.05 (0.59)	50	IC	Threat and deprivation	NA
Lewis-Morrarty et al. (2012)	United States	Comparison	44	5.02 (0.71)	49.2	CF	Deprivation	NA
Li et al. (2022)	United States	Correlation	141	15.05 (0.54); 16.07 (0.56); 16.48 (0.53)	47	IC	Deprivation	fMRI
Lynch et al. (2022)	United States	Comparison	873	39.5 (3.5); 41.2 (3.3)	52.7	CF, IC	Threat and deprivation	NA
Maxfield et al. (2023)	United States	Comparison	807	29.37 (3.82)	49	CF	Threat and deprivation	NA
Motsan et al. (2022)	Israel	Comparison	111	11.66 (1.22)	60.36	IC	Threat	ECG, RSP, cortisol level
Nikulina et al. (2013)	United States	Comparison	792	41.2 (3.3)	53	CF	Threat and deprivation	NA
Nweze et al. (2023)	United Kingdom	Comparison	2,965	24 (NA)	62.05	WM, IC	Threat and deprivation	NA
Pears et al. (2010)	United States	Comparison	117	4.45 (0.85)	47.5	IC	Threat and deprivation	NA
Savopuolus et al. (2022)	Australia	Correlation	615	10 (NA)	48.4	WM, CF, IC	Threat	NA
Tibu et al. (2016)	United States	Comparison	147	8.53 (NA)	52.73	WM, CF, IC	Deprivation	NA
Troller-Renfree et al. (2016)	United States	Comparison	85	11.81 (NA)	57.64	IC	Deprivation	EEG-ERP
Westermann et al. (2023)	German	Correlation	1,501	11.06 (0.92)	51.7	WM, CF, IC	Threat	NA
Yazgan et al. (2021)	United States	Correlation	395	16 (NA)	48	IC	Threat and deprivation	NA

*Note.* Age (years), sex (percentage of females) are given at CC measurement time. CC = cognitive control; WM = working memory; CF = cognitive flexibility; IC = inhibitory control; ACEs = adverse childhood experiences; NA = not available; EEG = electroencephalogram; ERP = event-related potential; ERSP = event-related spectral perturbation; fMRI = functional magnetic resonance imaging; ECG = electrocardiography; RSP = respiration/breathing rate and intensity.

The sample comprised 51% female and 49% male individuals, with the age span of participants in the studies included ranged from 2 to 41 years old (*M* = 13.41, *SD* = 8.5). The most studied participants were children and early adolescents, with limited studies examining the impact of ACEs on late adolescents and adults (see the histogram of studied age distribution in Supplemental Information 6).

Of 124 outcomes, 29 associations were captured for working memory, 17 for cognitive flexibility, and 78 for inhibitory control. The study quality assessment indicated a range from poor to good. One study (3.1%) had a poor quality, 21 studies (65.62%) had a fair quality, and 10 studies (31.25%) had a good quality score (see Supplemental Information 5 for a complete table of study quality assessment using the NOS).

### Overall Effect Sizes and Publication Bias

**Figure 2.**
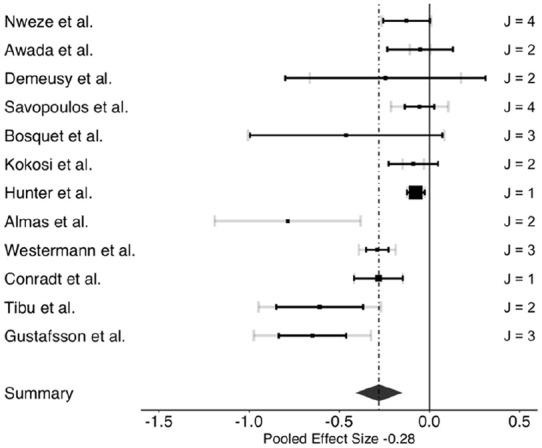
Forest plot of effect sizes of the association between ACEs and working memory. *Note*. ACEs = adverse childhood experiences.

**Figure 3.**
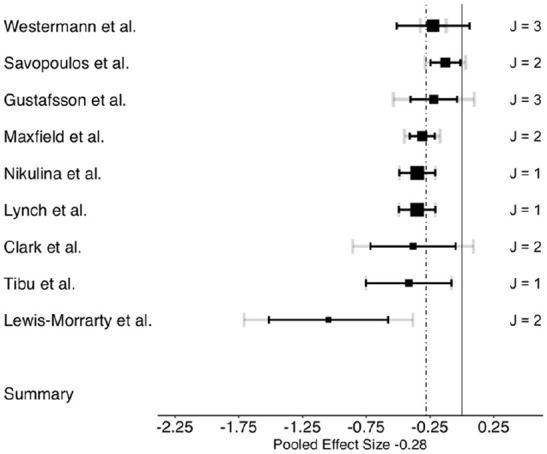
Forest plot of effect sizes of the association between ACEs and cognitive flexibility. *Note*. ACEs = adverse childhood experiences.

**Figure 4.**
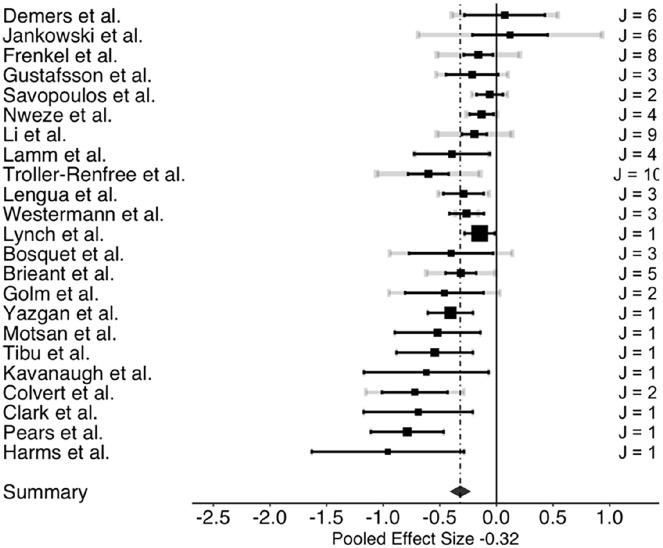
Forest plot of effect sizes of the association between ACEs and inhibitory control. *Note*. ACEs = adverse childhood experiences.

The three-level meta-analysis results indicated pooled estimates of the association between ACEs and overall cognitive control (*g* = −0.32; 95% confidence interval [CI: −0.40, −0.24]). Further, analysis revealed that ACEs negatively impacted all three cognitive control domains examined: working memory, cognitive flexibility, and inhibitory control. Table 2 presents the associations between ACEs and overall cognitive control, and each cognitive control domain: working memory, cognitive flexibility, and inhibitory control. The three-level meta-analysis results showed pooled estimates of the association between ACEs and working memory (*g* = −0.28; [−0.42, −0.14]), cognitive flexibility (*g* = −0.28; [−0.38, −0.19]), and inhibitory control (*g* = −0.32; [−0.42, −0.22]). Based on Cohen’s effect size interpretation (P. D. Ellis, 2010), the three associations had small-to-medium effect sizes. Figures 2 to 4 depict the dispersion of effect sizes from individual studies and the robustness of their respective evidence.

**Table 2.** Association Between ACEs and Each Cognitive Control Domain.

Cognitive Control Domains	No. of Studies	*k*	*g*	*SE*	95% CI	*I*^2^ (%)
Overall Cognitive Control	32	124	−0.32	0.04	[−0.40, −0.24]	84.42
Working Memory	12	29	−0.28	0.07	[−0.42, −0.14]	93.36
Cognitive Flexibility	9	17	−0.28	0.04	[−0.38, −0.19]	70.74
Inhibitory Control	17	78	−0.32	0.05	[−0.42, −0.22]	70.45

*Note. k =* number of effect sizes; *g* = effect size (Hedges’ *g*); *SE* = sampling error; CI = confidence interval; *I*^2^ = degree of heterogeneity; ACEs = adverse childhood experiences.

Heterogeneity was reported as significant for working memory (*Q*28 = 181.185; *p* < .0001; *I*^2^ = 93.36), cognitive flexibility (*Q*16 = 66.502; *p* < .0001; *I*^2^ = 70.74), and inhibitory control (*Q*77 = 193.538; *p* < .0001; *I*^2^ = 70.45).

**Figure 5.**
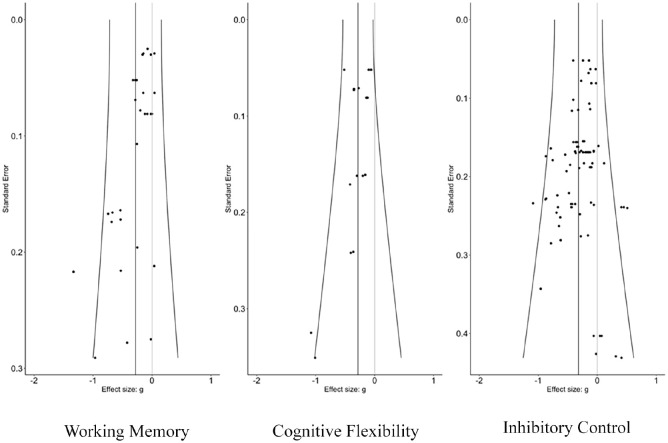
Funnel plot of effect sizes of the association between ACEs and each cognitive control domain. *Note*. ACEs = adverse childhood experiences.

When assessing small-study bias, Egger linear regression tests were conducted. A significant publication bias was observed, indicated by the *p*-values of .002 for working memory and .03 for cognitive flexibility. There was no notable indication of publication bias for inhibitory control, as indicated by the *p*-value of .39, suggesting the non-independence of effect sizes. Next, funnel plots were visually inspected for outcomes with a minimum of 10 cases. Figure 5 displays the funnel plots for working memory, cognitive flexibility, and inhibitory control. The overall effect sizes predominantly leaned toward the negative range, making the presence of publication bias less probable.

### Risk of Bias

Meta-regression was employed to assess the influence of risk of bias for each outcome with a minimum of 10 cases. The cumulative risk of bias ratings showed no significant correlation with the average effect size estimates across cognitive control domains—working memory: *F*(1, 27) = 1.31, *p* = .261; cognitive flexibility: *F*(1, 15) = 0.30, *p* = .588; inhibitory control: *F*(1, 76) = 0.18, *p* = .669. The collected evidence strongly supports the conclusion that there was no substantial association between the risk of bias rating and each cognitive control domain.

### Moderator Analyses

To investigate potential age-related moderation in the associations between ACEs and each cognitive control domain, a moderator analysis was carried out using age as a continuous variable. The results from the three-level moderator meta-analyses revealed that age did not moderate the relationships between ACEs and working memory, cognitive flexibility, and inhibitory control.

Moreover, for working memory, cognitive flexibility, and inhibitory control, no moderating effects were detected for the additional moderator variables investigated, which were study design (correlational study and comparative study), sex, and cognitive control measurement paradigms (e.g., digit span, flanker test, and trail making test). Notably for ACEs subtypes (threat, deprivation, or threat and deprivation), a moderation effect was found but only for cognitive flexibility, where a significant moderator effect of ACEs subtypes was found (*p* = .042). The effect size of the association between cognitive flexibility and deprivation (*g* = −0.70; 95% CI [−2.06, 0.64]) is higher than the association between cognitive flexibility and threat (*g* = −0.21; [−0.35, −0.08]) or combined threat and deprivation type of adversity (*g* = −0.33; [−0.44, −0.21]). The number of studies and effect sizes included in the moderator analysis for each cognitive control domain can be found in Supplemental Information 4. Detailed outcomes of the moderator analyses for working memory, cognitive flexibility, and inhibitory control are presented in Tables 3 to 6 respectively.

**Table 3.** Effects of Moderators on Working Memory.

Moderator	No. of Studies	*k*	*F*	*df*	*p*
Age	12	29	1.89	1, 27	.180
Sex	12	25	0.48	1, 23	.494
ACEs subtypes	12	29	3.17	2, 26	.059
CC task paradigms	12	29	0.35	3, 25	.789
Study type	12	29	1.4	1, 27	.250

*Note.* CC = cognitive control; *k =* number of effect sizes; *F* = the significance of the moderation effect; *df* = degree of freedom; ACEs = adverse childhood experiences.

**Table 4.** Effects of Moderators on Cognitive Flexibility.

Moderator	No. of Studies	*k*	*F*	*df*	*p*
Age	9	17	0.35	1, 15	.560
Sex	9	17	0.01	1, 15	.903
ACEs subtypes	**9**	**17**	**3.99**	**2, 14**	**.042**
CC task paradigms	9	17	0.44	3, 13	.728
Study type	9	17	4.20	1, 15	.058

*Note.* CC = cognitive control; *k =* number of effect sizes; *F* = the significance of the moderation effect; *df* = degree of freedom; ACEs = adverse childhood experiences; Bold values indicate a significant effect of the moderator on cognitive flexibility.

**Table 5.** Effect Sizes of the Association Between Each ACEs Subtype and Cognitive Flexibility.

ACEs Subtypes	No. of Studies	*k*	Hedges’ *g*	95% CI
Threat	4	10	−0.21	[−0.35, 0.08]
Deprivation	2	3	−0.70	[−2.06, 0.64]
General	3	4	−0.33	[−0.44, −0.21]

*Note. k =* number of effect sizes; Hedges’ *g* = effect size (Hedges’ *g*); CI = confidence interval; ACEs = adverse childhood experiences.

**Table 6.** Effects of Moderators on Inhibitory Control.

Moderator	No. of Studies	*k*	*F*	*df*	*p*
Age	23	78	2.80	1, 76	.098
Sex	23	78	1.71	1, 76	.194
ACEs subtypes	23	78	0.377	2, 75	.686
CC task paradigms	23	78	0.81	5, 72	.542
Study type	23	78	0.01	1, 76	.930

*Note.* CC = cognitive control; *k =* number of effect sizes; *F* = the significance of the moderation effect; *df* = degree of freedom; ACEs = adverse childhood experiences.

## Discussion

This systematic review and meta-analysis investigated the association between ACEs and cognitive control functions across ages. In this study, a broad and inclusive definition of ACEs was adopted to capture the wide range of adversities that individuals may face during childhood, such as various forms of abuse, neglect, household dysfunction, financial crisis, and mental illness of caregivers.

It was found that ACEs had an equivalent adverse impact on all three distinct domains of cognitive control, working memory, cognitive flexibility, and inhibitory control in childhood, adolescence, and adulthood. In all three cognitive control domains, this effect was not moderated by age, study type (correlation and comparison), sex, and the cognitive control task paradigm used. While only for cognitive flexibility, but not working memory and inhibitory control, ACEs subtypes were observed to moderate the impact of ACEs, specifically deprivation subtype of ACEs has a stronger negative association with cognitive flexibility, compared to threat and threat and deprivation subtypes of ACEs.

### The Negative Impact of ACEs on All Cognitive Control Domains

Our study found that higher levels of ACEs were consistently associated with diminished cognitive control in children, adolescents, and adult participants. These results align with prior meta-analysis studies that similarly documented the adverse impact of ACEs on cognitive control in children and adolescents (Johnson et al., 2021; Op Den Kelder et al., 2018). Importantly, our study extends the existing literature by providing evidence that ACEs also negatively impact cognitive control in adulthood. This finding highlights the long-term consequences of ACEs, emphasizing the critical importance of addressing the impact of ACEs across the lifespan.

Moreover, it is imperative to underscore that, in this study, the collective effect sizes representing the influence of ACEs on the three cognitive control functions ranged from small to medium magnitude. Comparatively, when juxtaposed with a similar meta-analysis encompassing both cross-sectional and longitudinal studies (Johnson et al., 2021; Op Den Kelder et al., 2018), this current study unveiled slightly smaller effect sizes. While the outcomes underscore a significant negative impact of ACEs, focusing solely on longitudinal or prospective studies in this meta-analysis may imply that the extended intervals between ACEs measurement and cognitive control outcomes could facilitate the occurrence of intervening factors. These factors might moderate the effect of ACEs on cognitive control, potentially accounting for the observed smaller effect sizes. Furthermore, it suggests that the negative impact might not manifest uniformly across all individuals experiencing ACEs in the studies examined here.

We did not find detailed information in the included studies regarding potential intervening factors that could have influenced the effects of ACEs on cognitive control, either positively or negatively. Therefore, our study did not allow for an examination of potential protective elements such as benevolent experiences, educational attainment, and early interventions. Future research endeavors should broaden their scope to include the measurement of a wider range of contextual and biological factors, including genetic data. This approach will facilitate a more comprehensive understanding of how these factors may influence the developmental impact of ACEs exposure on cognitive control.

It is also possible that the effect sizes which were higher in Johnson et al. (2021) and Op Den Kelder et al. (2018) may have been overinflated because of bias due to the inclusion criteria. Both studies combined cross-sectional and longitudinal studies in their meta-analysis, which could be a potential result bias since relating cross-sectional data to infer effects could hinder causal inferences. Additionally, some studies included in their meta-analyses used self-report or teacher/caregiver reports to measure cognitive control. This could be an issue because there is a very low correlation between self-report and behavioral measurement of cognitive control, particularly in the inhibition domain (Wennerhold & Friese, 2020). Therefore, it is advisable not to compare studies using self-report and behavioral measurement since self-report and behavioral tasks target distinct aspects of the theoretical framework of cognitive control (Wennerhold & Friese, 2020). Apart from this, when contrasted with self-reporting, behavioral tasks are specifically designed to objectively evaluate performance and measure narrower, more domain-specific facets of cognitive control.

It is important to note that although not significant, the effect size for the impact of ACEs on inhibitory control was stronger than working memory and cognitive flexibility. From a theoretical perspective, inhibitory control might be particularly vulnerable to the effects of ACEs due to its critical role in regulating behavior and emotions, which are often disrupted in individuals with high exposure to ACEs (Bartholomew et al., 2021). Inhibitory control is essential for suppressing inappropriate responses, managing impulses, and exerting self-control. These functions are heavily influenced by stress and trauma (Sætren et al., 2023; Van Der Bij et al., 2020), which are central components of ACEs. Moreover, the PFC, which is primarily responsible for inhibitory control, is highly susceptible to the adverse effects of ACEs (Gee & Casey, 2015; McEwen, 2007). However, this observed difference in total effect size could also be explained by the greater number of studies and effect sizes resulting from studies examining the association with inhibitory control incorporated in the analysis, compared to studies of working memory and cognitive flexibility. Consequently, the smaller number of studies on working memory and cognitive flexibility may limit the ability to detect significant associations with ACEs in these domains (Button et al., 2013), potentially skewing the apparent strength of the relationship toward inhibitory control.

### The Negative Impact of ACEs on Cognitive Control Across the Lifespan

Another significant finding derived from the meta-analyses conducted in this study pertains to the moderator analysis, which indicated that age did not moderate the negative effect of ACEs on cognitive control abilities. Irrespective of the individual’s age, ACEs consistently and similarly impacted cognitive control. This finding suggests that the negative association between ACEs and cognitive control shown across the lifespan. In essence, ACEs experienced during childhood detrimentally affect cognitive control development early on in childhood, also in adolescence and adulthood. This underscores the importance of targeted interventions and support mechanisms aimed at mitigating the long-term impact of ACEs on cognitive functioning across the lifespan. However, the non-significant moderating effect of age could also be attributed to the relatively small number of studies on this association in adulthood (five studies, *k* = 14). This limited sample size might reduce the statistical power to detect a moderating effect and increases the likelihood of Type II errors, where true effects may go unnoticed (Button et al., 2013).

Notably in this study, we examined the impact of chronological age on the effect of ACEs on cognitive control, hence it remains unknown whether other developmental factors, such as puberty, might influence how ACEs affect cognitive control, particularly in adolescents. Recent research suggests that experiencing high levels of ACEs can lead to an early onset of puberty (Colich, Platt, et al., 2020; Colich, Rosen, et al., 2020), and early puberty is linked to poorer cognitive control, notably inhibitory control ability (Deater-Deckard et al., 2019). Furthermore, accelerated puberty timing mediates the negative relationship between ACEs and psychopathological symptoms in adolescents, including anxiety, depression, social withdrawal, and aggressive behavior (Petrican et al., 2021). Petrican et al.’s study implies that ACEs result in accelerated pubertal maturation, subsequently leading to a greater risk of experiencing psychopathological symptoms in adolescence. These research findings highlight the intricate connections between ACEs, biological changes like puberty, and well-being during adolescence, emphasizing the multifaceted challenges faced by adolescents exposed to ACEs. Therefore, it is crucial to consider developmental factors like puberty when investigating how ACEs impact cognitive control in adolescents.

Future research exploring the underlying mechanisms linking ACEs exposure, pubertal timing, and cognitive control could offer deeper insights into the complex pathways through which ACEs affect adolescents. Moreover, evidence on the effect of ACEs on cognitive control performance, and how puberty timing influences the effect in adolescents is scarce and needs further exploration. Such findings may inform more targeted interventions and preventive strategies, ultimately enhancing our ability to support adolescents in overcoming the long-term consequences of ACEs.

Notably, our systematic review did not uncover any studies that specifically examined individuals older than 41 years or focused on older age cohorts. Hence, another unresolved question for future research pertains to the influence of ACEs on cognitive control among the elderly. There is a possibility that ACEs could contribute to a more pronounced decline in neurocognitive function during old age.

In this study, we also looked at the impact of biological sex in moderating the negative association but found no such association. This, interestingly, contrasts with ACEs impact studies, which show key biological sex differences. For example, research indicates that women who have experienced ACEs are more likely to have moderate to severe mental distress, whereas men who experienced ACEs are more likely to exhibit suicidal tendencies, self-harm, and substance abuse (Jones et al., 2022). The intriguing dissociation between the lack of moderating effect of sex on the association between ACEs and cognitive control, despite existing evidence of sex differences in the impact of ACEs on mental health outcomes, indicates intricate interactions between ACEs, sex, and psychological functioning. While sex disparities have been documented in outcomes such as mental distress and substance use disorders resulting from ACEs, the consistent adverse impact of ACEs on cognitive control regardless of sex indicates that cognitive functioning may be influenced by ACEs through mechanisms independent of sex. This discrepancy underscores the multifaceted nature of ACEs effects and emphasizes the need for further investigation into the underlying mechanisms that drive differential outcomes. Understanding why sex differences emerge in some contexts but not others could provide valuable insights for designing more effective interventions that address the diverse consequences of ACEs on mental health.

### Impact of Study Design, ACEs Dimensions, and Cognitive Control Task Paradigms on the Association Between ACEs and Cognitive Control

We found that neither the study design (correlational vs. comparative) nor the type of cognitive control task paradigms used significantly moderated the effect of the negative association between ACEs and working memory, cognitive flexibility, and inhibitory control. This suggests that the detrimental impact of ACEs on working memory, cognitive flexibility, and inhibitory control remains consistent across different study methodologies and task variations. These findings underscore the strength of the relationship between ACEs and cognitive control deficits, indicating that it transcends methodological differences and specific task demands.

In the context of the cognitive control task paradigm, further categorization of cognitive control could have been used for moderator analysis, particularly in distinguishing between “hot” and “cool” cognitive control. However, within the context of our meta-analysis, only one study employed what could potentially be classified as a “hot” cognitive control measure (i.e., a go/no-go task using emotional picture stimuli). “Hot” cognitive control refers to the regulation of behavior in emotionally charged situations, where managing emotions and impulses is crucial (Zelazo, 2020; Zelazo et al., 2024). This type of cognitive control is typically activated in contexts involving immediate rewards or consequences, requiring individuals to effectively navigate emotional responses (Zelazo et al., 2024). Conversely, “cool” cognitive control involves regulating behavior in more abstract and decontextualized situations, which lack immediate emotional stakes (Zelazo, 2020; Zelazo & Carlson, 2012). This form of cognitive control is primarily engaged in tasks that require logical reasoning, planning, and problem-solving, independent of strong emotional influences (Zelazo & Carlson, 2012).

Due to the limited data available in this study, it is not feasible to conduct a robust moderator analysis to examine the differential effects of ACEs on these subtypes of cognitive control. While we acknowledge the potential importance of distinguishing between “hot” and “cool” cognitive control, the current dataset does not allow for a comprehensive exploration of these differences. Therefore, where feasible, we recommend that future meta-analysis in this topic conduct moderator analysis on “hot” and “cool” cognitive control, as some prior research indicates that ACEs may differentially affect these two distinct forms of cognitive control (e.g., Cao et al., 2023; Sturge-Apple et al., 2017; Sun et al., 2023).

However, it is worth noting that, while the classification of “hot” and “cool” cognitive control provides a useful framework for differentiating between affective and non-affective forms of cognitive control, it is important to recognize that this distinction can sometimes be overly simplistic (Pessoa, 2008; Zelazo & Carlson, 2012). The interaction between emotional and cognitive processes is inherently complex, and the categorization into “hot” and “cool” subtypes may not fully capture the spectrum of cognitive control processes (Gray, 2004; Pessoa, 2008).

A key aspect which we explored in this study was the potential role of different subtypes of ACEs in moderating the association between ACEs and each cognitive control domain—working memory, cognitive flexibility, and inhibitory control. Based on the DMAP (McLaughlin & Sheridan, 2016), which highlights the varying effect of specific ACEs subtypes (threat and deprivation) on neurobiological outcomes, our moderator analysis explored how variations in ACEs subtypes may affect cognitive control. Surprisingly, our threat and deprivation ACEs subtypes did not act as moderators for the association between ACEs and working memory and inhibitory control abilities. Findings are inconsistent with the results of the meta-analysis study by Johnson et al. (2021), who found a moderating effect of ACEs subtypes, emphasizing greater associations of deprivation compared to threat with working memory and inhibitory control. This discrepancy could be attributed to Johnson et al.’s inclusion of retrospective and prospective studies, where retrospective reports may introduce memory-related confounds (Baldwin et al., 2019; Radford et al., 2013). On the other hand, this current study relying on prospective self-reports suggests that variations in results may stem from potential differences in reporting accuracy.

Notably, this study did find a significant moderation effect of ACEs subtypes on cognitive flexibility, such that the ACEs subtype of deprivation exhibited a stronger negative association with cognitive flexibility compared to threat and threat and deprivation subtypes. This result is consistent with Op Den Kelder et al.’s (2018) meta-analysis, which also suggests that cognitive flexibility may exhibit heightened susceptibility to the impact of deprivation-type ACEs. This observation may be attributed to the nature of deprivation experiences, characterized by prolonged exposure to environmental stressors and restricted access to resources vital for cognitive development (McLaughlin, Sheridan, & Lambert, 2014). Such prolonged adversity may impair the development and adaptation of cognitive flexibility skills, which are crucial for adjusting to changing environmental demands and problem-solving. Yet, it is important to note that future research and replication are needed, as the work of Johnson et al. (2021) does not support our findings. Although this current study, Op Den Kelder et al. (2018) and Johnson et al. (2021) utilize the same classification of ACEs subtypes based on the DMAP (McLaughlin & Sheridan, 2016). Johnson et al. (2021) reported no moderating effect of ACEs subtypes on cognitive flexibility. Rather, they found a moderating effect of ACEs subtypes in working memory and inhibitory control. The inconsistency and lack of significant findings regarding the moderation effect of ACEs subtypes could also be due to the categorization of ACEs subtypes, that is, threat and deprivation. This categorization may not accurately capture the true complexity of adversity across various studies included in the meta-analysis.

Indeed, defining a clear dimension model or the subtypes of ACEs is a complex challenge. Some researchers question the validity of established ACEs subtypes like threat and deprivation, arguing that these categorizations often yield inconsistent results (Pollak & Smith, 2021; Smith & Pollak, 2021). Pollak and Smith (2021) raise doubts about the robustness and accuracy of these concepts in explaining the mechanisms of ACEs’ impact, especially concerning complex biological responses resulting from ACEs exposure. For example, children exposed to parental abuse are frequently classified under the threat dimensions, even though in an abusive home real-life situation, they also experience deprivation due to the simultaneous lack of security, comfort, and protection. In fact, exposure to threat and deprivation often heavily overlap, making it challenging to distinguish their distinct impact. ACEs are multidimensional in factual circumstances and often coincide in complicated ways. Notably, there is a lack of neurobiological evidence supporting the differentiation between threat and deprivation (Smith & Pollak, 2021).

In light of this, exploring more nuanced models of ACEs subtypes holds promise for advancing our understanding of adversity and its diverse impacts on individuals, emphasizing the importance of comprehensive frameworks in future research endeavors. Some researchers have proposed alternative models to represent ACEs subtypes. For instance, the environmental experience integrated model (B. J. Ellis et al., 2022) offers a more specific categorization, distinguishing between harshness stemming from threats, harshness stemming from deprivation, and the unpredictable nature of the environment. There is also a model that specifically differentiates the deprivation dimension into material and emotional types (Dennison et al., 2019). In addition, another alternative way forward is not to use categorization, but rather another approach using a data-driven method that could be used to study the dimensions of ACEs. When using the data-driven approach, the decision on organizing and recognizing patterns of ACEs dimension is based on the data and not solely established by the researcher, and it is suggested as one appropriate method to generate a more precise dimension of ACEs (Gee et al., 2021).

### Study Limitations

A key limitation of this study is the relatively limited research (*n* = 32) included in the meta-analyses, which may have restricted the statistical power. Our study found a significant small number of studies publication bias; therefore, consideration should be given to publication bias when analyzing the findings of this study, as it could potentially inflate the effect sizes, indicating possibly understated reported estimates. This implies that the actual impact of ACEs and the influence of moderator variables could be more significant than what is suggested by the observed data, but due to the cautious approach taken in the study’s analysis, the reported results might underestimate the true extent of the effect. This limitation emphasizes the need for more research incorporating longitudinal data to be included in the meta-analysis to explore the impact of ACEs on cognitive control more comprehensively.

It is also acknowledged that the conceptualization of adverse experience in this study is broad and inclusive to ensure that the analysis encompasses the full spectrum of ACEs, thus providing a more holistic understanding of their impact on cognitive control. However, this broad conceptualization may come with limitations. By including studies with different methods of measuring ACEs, there may be variability in how ACEs are reported and recorded. This variability can introduce heterogeneity in the analysis, potentially affecting the comparability of results across studies (Baldwin et al., 2019; Lacey et al., 2022; McLennan et al., 2020). To address the potential challenges associated with a broad range of ACEs measurements used across the included studies, this study categorized adverse experiences using the DMAP (McLaughlin & Sheridan, 2016), providing a more structured framework for the analysis. This method enables us to account for potential variations in effect sizes due to different types of ACEs and to identify which subtypes may have the most significant impact on cognitive control. This approach helps in highlighting specific patterns of adversity and their unique impacts on cognitive control (McLaughlin, Sheridan, & Lambert, 2014). Despite these efforts, we acknowledge that the heterogeneity in ACEs measures is a limitation. Future research would benefit from more standardized definitions and consistent measurement tools to enhance comparability across studies. This would enable a more precise understanding of how different types of ACEs influence cognitive control and other outcomes.

Another limitation of this study pertains to the significant heterogeneity observed in the pooled estimates of effect sizes for working memory and inhibitory control. Significant heterogeneity indicates that the study’s results for working memory and inhibitory control varied a lot, which made it harder to draw clear conclusions. Consequently, it is essential to approach the results of this study with caution due to the substantial variations within the data. To address this heterogeneity, moderator analyses were conducted to identify its contributing factors. These encompassed various article characteristics, including sample features, study design, ACEs subtypes, and cognitive control measurement paradigms. Additionally, the use of three-level meta-analyses was intended to address both between-study and within-study variances.

However, it was found that except for ACEs subtypes, none of these moderator variables had an influence on the magnitude of effect sizes across all cognitive control domains. This suggests that there may be other unexamined variables not covered in this study that could affect the longitudinal association between ACEs and cognitive control. The substantial heterogeneity in these associations might also stem from differences in the timing, duration, and severity of ACEs, or various protective factors experienced by the participants across various studies. It is important to note that many of the studies in this current meta-analysis did not measure the timing, duration, and severity of ACEs. As a result, it was not possible to code out these variables and test them as a meaningful moderator in the meta-analysis. Nonetheless, it is imperative that future research explores these aspects to gain a more comprehensive understanding of the effects of ACEs on development.

Furthermore, it’s worth noting that neurobiological characteristics, such as pubertal maturation, stress reactivity, and positive or benevolent experiences following exposure to ACEs, may have also contributed to the observed heterogeneity. Hence, it is recommended that future studies directly evaluate these possible sources of variation and heterogeneity, offering a more thorough comprehension of the variances in the strength of associations between and within adversity experiences.

Finally, a key limitation of this study is the inclusion of studies based solely on Western samples. While our inclusion criteria did not explicitly focus on Western samples, our search was constrained by the language criterion, which was limited to English language publications. Consequently, studies based on non-Western samples published in languages other than English may not have been captured in our search. This limitation introduces potential bias due to language restrictions, which could have led to the inadvertent exclusion of relevant studies from non-Westerns contexts. As a result, our findings may not fully generalize to non-Western populations, limiting the external validity of the study. There is also a possibility that the unavailability of non-Western studies also reveals a significant gap in the scientific study of this research question in the non-Western samples. Moving forward, we advocate for future research to adopt a more inclusive approach that encompasses diverse cultural and geographical contexts.

### Implications for Research, Practice, and Policy

The results of this study offer significant insights for research, practice, and policymaking. First, it is critical to elaborate on the research and theoretical implications of this study. This meta-analysis found the longitudinal association between ACEs and each domain of cognitive control across the lifespan, and most of the moderator variables did not influence the effect sizes found. Future research is needed to directly evaluate the potential sources of variation to elucidate the variances in the strength of association both between and within exposure to ACEs, thus providing evidence for risk and protective factors that may alleviate the adverse impact of ACEs. Further study is also needed to elucidate the mechanisms through which neurobiological risk and protective factors such as pubertal maturation may contribute to the effect of ACE exposure on cognitive control, which may, in turn, be associated with the emergence of psychological disorders.

Within the selection of eligible studies in this systematic review, a noticeable gap was identified in the range of age groups that had been investigated. While a significant body of research focused on early childhood and adolescence (ages 0–14), there was a limited representation of studies encompassing late adolescence and adulthood (ages >15). This gap in the developmental stages studied suggests that future research should aim to fill this void and explore the impact of ACEs on cognitive control during late adolescence and older adulthood/old age. This would provide additional evidence and insights into how ACEs affect cognitive control during this particular developmental stage.

While this meta-analysis reveals the impact of ACEs using the dimensional perspective, which recognizes ACEs along a spectrum rather than as discrete categories, it is recommended that future studies consider not only dimensional but also incorporate the cumulative and interactive perspectives. This could provide further insights into the nuanced effects of ACEs. The cumulative perspective, which considers the accumulation of ACEs over time, emphasizes the importance of understanding how the total burden of adversity interacts with a specific developmental period (Evans et al., 2013; Masten & Cicchetti, 2010; Shonkoff et al., 2012). Furthermore, the interactive perspective underscores the dynamic interplay between individual susceptibilities and environmental factors, offering a more comprehensive framework for exploring differential outcomes (Rutter, 2012; Shonkoff et al., 2012). These perspectives are essential for advancing the theoretical landscape in this field, providing more robust models that can better capture the complexity of childhood adversity and its effects on cognitive control.

Reflecting on this current study’s methodological strengths and limitations, it is crucial to emphasize methodological rigor in future research. Ensuring the inclusion of diverse and representative samples is vital for reducing biases related to demographic homogeneity and improving the generalizability of findings. Moreover, the standardization of measures across studies should be a priority. Variability in how ACEs and cognitive control are operationalized can introduce significant heterogeneity, making it difficult to compare results. Utilizing consistent definitions and validated measures across studies would reduce this variability and allow for more accurate comparisons in meta-analytic review.

Another crucial methodological consideration for meta-analyses is the careful selection of moderator variables. For instance, as discussed earlier, distinction between “hot” and “cool” cognitive control, as well as cumulative and interactive perspective of ACEs, could be explored as moderators to uncover differential effects. Lastly, the field would benefit from addressing publication bias by including gray literature and unpublished studies, thereby providing a more comprehensive and less biased understanding of the effects of ACEs. By focusing on these methodological improvements, future meta-analyses can provide more precise, reliable, and generalizable findings that will advance the field of ACEs research.

Next, in addition to research and theoretical implications, this current study could offer practical recommendations. The findings suggested that the negative impact of ACEs on all cognitive control domains can be manifested early or later in life. Therefore, the results underscore the importance of prevention and early intervention initiatives aimed at decreasing exposure to ACEs and the negative consequences of ACEs. Early prevention and intervention programs aimed at enhancing cognitive control abilities may mitigate the adverse effects of ACEs and facilitate the recovery of individuals exposed to ACEs, considering the prominent role cognitive control plays in improving various developmental abilities (Ahmed et al., 2019; Blair & Raver, 2015; Moffitt et al., 2011). For instance, given that a slightly greater effect is observed on the inhibitory control domain, interventions targeting this specific cognitive control ability could be prioritized. These interventions may include inhibitory control training programs, mindfulness-based interventions, or cognitive behavioral therapies designed to enhance inhibitory control abilities in individuals exposed to ACEs (Cásedas et al., 2020; Dhami et al., 2022; Jiao et al., 2021). Moreover, this study found that deprivation appears to have a more negative impact on cognitive flexibility compared to other ACEs subtypes. In light of these findings, interventions aimed at addressing deprivation-related ACEs, such as for institutionalized children, could focus on enhancing cognitive flexibility skills through targeted interventions such as cognitive-behavioral therapy, educational interventions, or social support programs. Children living in institutionalized care may benefit from cognitive flexibility since it may increase their resilience and socioemotional adjustment (Khambati et al., 2018).

Finally, concerning policy implications, this systematic review and meta-analysis add to the extensive empirical evidence on the impact of ACEs on cognitive control, emphasizing its critical importance. Policymakers should prioritize developing and enforcing policies or legislation aimed at preventing ACEs and mitigating their impact on cognitive control. This includes allocating resources, prioritizing funding, and expanding access to high-quality early childhood education, parenting support programs, and trauma-informed mental health services with intervention specifically target cognitive control deficits associated with ACEs, namely working memory, cognitive flexibility, and inhibitory control.

Furthermore, investment in future longitudinal research on ACEs’ long-term impact on cognitive control may provide additional insights into effective intervention strategies and preventive measures. A better understanding of how ACEs influence cognitive control abilities over time can guide further policy decisions and resource allocation to support individuals affected by ACEs across the lifespan.

## Conclusion

This systematic review and meta-analysis quantitatively synthesized primary research on the longitudinal association between ACEs and cognitive control functions, namely working memory, cognitive flexibility, and inhibitory control. ACEs exposure was found to be associated with a reduction of each cognitive control domain across the lifespan, and none of the mediator variables examined, age, sex, ACEs subtypes, cognitive control measurement paradigm, and study design, impacted the association found between ACEs and working memory and inhibitory control. ACEs subtypes were found to influence the association between ACEs and cognitive flexibility. Cultivating awareness of the pervasive influence of ACEs on cognitive control functions is imperative for designing targeted interventions and support systems. By recognizing the lasting impact of ACEs, policymakers, educators, and healthcare professionals can work collaboratively to develop evidence-based strategies that empower individuals affected by childhood adversity, promoting better cognitive control outcomes and overall well-being. Acknowledging the necessity for deeper exploration, future research initiatives are encouraged to investigate potential mediator and moderator variables, aiming to elucidate the variations in the strength of associations between ACEs and cognitive control in specific contexts. This endeavor is crucial for understanding the underlying mechanisms linking ACEs to cognitive control. Longitudinal studies examining the interplay between specific ACEs exposure, biological factors like pubertal timing, and specific cognitive control deficits have the potential to provide invaluable insights.

**Table:** Summary of Critical Findings.

1. ACEs are associated with poor working memory (*g* = −0.28) 2. ACEs are associated with poor cognitive flexibility (*g* = −0.28) 3. ACEs are associated with poor inhibitory control (*g* = −0.32) 4. Age, sex, cognitive control task paradigms, and study design did not moderate the association between ACEs and working memory, cognitive flexibility, and inhibitory control 5. ACEs subtypes moderated the association between ACEs and cognitive flexibility but not working memory and inhibitory control

*Note*. ACEs = adverse childhood experiences.

**Table:** Summary of Implications of the Review for Practice, Policy, and Research.

Research	Future research should examine other potential moderating factors that could act as protective or additional risk factors for impaired cognitive control.
Practice	The findings underscore the importance of early prevention and intervention initiatives aimed at decreasing exposure to ACEs and their negative consequences, particularly targeting cognitive control abilities, with prioritized interventions focusing on inhibitory control, and specifically targeted cognitive flexibility skills for deprivation at-risk population.
Policy	The findings emphasize the critical need for government prioritization of policies aimed at mitigating and preventing ACEs, with targeted resources for early childhood education, parenting support programs, and trauma-informed mental health services addressing specific cognitive control deficits associated with ACEs, including inhibitory control, cognitive flexibility, and working memory, while also recognizing the heightened impact of deprivation on cognitive flexibility.

*Note*. ACEs = adverse childhood experiences.

## Supplemental Material

sj-docx-1-tva-10.1177_15248380241286812 – Supplemental material for The Impact of Adverse Childhood Experiences on Cognitive Control Across the Lifespan: A Systematic Review and Meta-analysis of Prospective Studies Supplemental material, sj-docx-1-tva-10.1177_15248380241286812 for The Impact of Adverse Childhood Experiences on Cognitive Control Across the Lifespan: A Systematic Review and Meta-analysis of Prospective Studies by Satwika Rahapsari and Liat Levita in Trauma, Violence, & Abuse
