# Supplementary material for: The Impact of Adverse Childhood Experiences on Cognitive Control Across the Lifespan: A Systematic Review and Meta-analysis of Prospective Studies
Source: Trauma Violence Abuse. 2024 Oct 13;26(4):712–33. doi: 10.1177/15248380241286812 (PMC12397554; doi:10.1177/15248380241286812)

**Supplementary Information 1**

*Search Strategy*

| **Concept** | **Terms used** |
| --- | --- |
| Exposure: Adverse Childhood Experiences (ACEs) | “adverse child* experience*” OR “child* adversit*” OR “early life adversit*” OR “early-life stress” OR “child* abus*” OR “child* maltreat*” OR “child* neglect” OR “child* trauma” OR “development* trauma” OR “psychological trauma” OR “emotional trauma” OR “traumatic experience*” OR “domestic violence” OR “family violence” OR “emotion* abuse” OR “physical abuse” OR “verbal abuse” OR “sex* abuse” OR “adoption” OR “foster care” OR “institutionalization” OR “institutional rearing” OR “food insecurity” OR “bully*” OR “victimization” OR “peer victimi*” OR “peer violence” OR “community violence” OR “neighbourhood violence” OR “war*” OR “ethnic cleansing” OR “genocid*” OR “terrorism” OR “unaccompanied minor” OR “refugee*” OR “asylum*” OR “disaster victim*” OR “disaster*” |
| Outcome: Cognitive Control | “cognitive control” OR “executive control” OR “executive function*” OR “effortful control” OR “attentional control” OR “interference control” OR “interference effect*” OR “self-control” OR “self-regulati*” OR “inhibitory control” OR “inhibition” OR “disinhibition” OR “response inhibition” OR “reactive inhibition” OR “proactive inhibition” OR “selective attention” OR “focused attention” OR “sustained attention” OR “divided attention” OR “response monitoring” OR “error monitoring” OR “error processing” OR “conflict monitoring” OR “conflict adaptation” OR “working memory” OR “updating” OR “continuous working memory” OR “cognitive flexibility” OR “shifting” |
| Study design | “cohort stud*” OR “prospective cohort stud*” OR “prospective stud*” OR “prospect*” OR “longitud*” OR “longitudinal stud*” |

**Supplementary Information 2**

*Reference List of the Included Articles for Meta-analysis*

​​Almas, A. N., Degnan, K. A., Nelson, C. A., Zeanah, C. H., & Fox, N. A. (2016). IQ at age 12 following a history of institutional care: Findings from the Bucharest Early Intervention Project. *Developmental Psychology*, *52*(11), 1858–1866. <https://doi.org/10.1037/dev0000167>

Awada, S. R., Shelleby, E. C., Alfonso, D., & Keane, J. (2023). Differential Mechanisms Linking Early Childhood Threat and Social/Environmental Deprivation to Adolescent Conduct Problems. *Journal of Family Violence*. <https://doi.org/10.1007/s10896-023-00627-2>

Bosquet Enlow, M., Petty, C. R., Svelnys, C., Gusman, M., Huezo, M., Malin, A., & Wright, R. J. (2019). Differential Effects of Stress Exposures, Caregiving Quality, and Temperament in Early Life on Working Memory versus Inhibitory Control in Preschool-Aged Children. *Developmental Neuropsychology*, *44*(4), 339–356. <https://doi.org/10.1080/87565641.2019.1611833>

Brieant, A., Clinchard, C., Deater-Deckard, K., Lee, J., King-Casas, B., & Kim-Spoon, J. (2023). Differential Associations of Adversity Profiles with Adolescent Cognitive Control and Psychopathology. *Research on Child and Adolescent Psychopathology*, *51*(12), 1725–1738. <https://doi.org/10.1007/s10802-022-00972-8>

Clark, H. M., Grogan-Kaylor, A. C., Galano, M. M., Stein, S. F., & Graham-Bermann, S. A. (2022). Preschoolers’ Intimate Partner Violence Exposure and Their Speeded Control Abilities Eight Years Later: A Longitudinal Mediation Analysis. *Journal of Interpersonal Violence*, *37*(19–20), NP18496–NP18523. <https://doi.org/10.1177/08862605211035883>

Colvert, E., Rutter, M., Kreppner, J., Beckett, C., Castle, J., Groothues, C., Hawkins, A., Stevens, S., & Sonuga-Barke, E. J. S. (2008). Do Theory of Mind and Executive Function Deficits Underlie the Adverse Outcomes Associated with Profound Early Deprivation?: Findings from the English and Romanian Adoptees Study. *Journal of Abnormal Child Psychology*, *36*(7), 1057–1068. <https://doi.org/10.1007/s10802-008-9232-x>

Conradt, E., Abar, B., Lester, B. M., LaGasse, L. L., Shankaran, S., Bada, H., Bauer, C. R., Whitaker, T. M., & Hammond, J. A. (2014). Cortisol Reactivity to Social Stress as a Mediator of Early Adversity on Risk and Adaptive Outcomes. *Child Development*, *85*(6), 2279–2298. <https://doi.org/10.1111/cdev.12316>

Demers, L. A., Hunt, R. H., Cicchetti, D., Cohen-Gilbert, J. E., Rogosch, F. A., Toth, S. L., & Thomas, K. M. (2022). Impact of childhood maltreatment and resilience on behavioral and neural patterns of inhibitory control during emotional distraction. *Development and Psychopathology*, *34*(4), 1260–1271. <https://doi.org/10.1017/S0954579421000055>

Demeusy, E. M., Handley, E. D., Rogosch, F. A., Cicchetti, D., & Toth, S. L. (2018). Early Neglect and the Development of Aggression in Toddlerhood: The Role of Working Memory. *Child Maltreatment*, *23*(4), 344–354. <https://doi.org/10.1177/1077559518778814>

Frenkel, T. I., Donzella, B., Frenn, K. A., Rousseau, S., Fox, N. A., & Gunnar, M. R. (2020). Moderating the Risk for Attention Deficits in Children with Pre-Adoptive Adversity: The Protective Role of Shorter Duration of out of Home Placement and Children’s Enhanced Error Monitoring. *Journal of Abnormal Child Psychology*, *48*(9), 1115–1128. <https://doi.org/10.1007/s10802-020-00671-2>

Golm, D., Sarkar, S., Mackes, N. K., Fairchild, G., Mehta, M. A., Rutter, M., ERA Young Adult Follow Up study team, & Sonuga-Barke, E. J. (2021). The impact of childhood deprivation on adult neuropsychological functioning is associated with ADHD symptom persistence. *Psychological Medicine*, *51*(15), 2675–2684. <https://doi.org/10.1017/S0033291720001294>

Gustafsson, H. C., Coffman, J. L., & Cox, M. J. (2015). Intimate partner violence, maternal sensitive parenting behaviors, and children’s executive functioning. *Psychology of Violence*, *5*(3), 266–274. <https://doi.org/10.1037/a0037971>

Harms, M. B., Birn, R., Provencal, N., Wiechmann, T., Binder, E. B., Giakas, S. W., Roeber, B. J., & Pollak, S. D. (2017). Early life stress, FK506 binding protein 5 gene ( *FKBP5* ) methylation, and inhibition-related prefrontal function: A prospective longitudinal study. *Development and Psychopathology*, *29*(5), 1895–1903. <https://doi.org/10.1017/S095457941700147X>

Hunter, C. L., & Shields, G. S. (2022). Mediators of the associations between family income during adolescence and adult long-term memory and working memory. *Cognitive Development*, *61*, 101140. <https://doi.org/10.1016/j.cogdev.2021.101140>

Jankowski, K. F., Bruce, J., Beauchamp, K. G., Roos, L. E., Moore, W. E., & Fisher, P. A. (2017). Preliminary evidence of the impact of early childhood maltreatment and a preventive intervention on neural patterns of response inhibition in early adolescence. *Developmental Science*, *20*(4), e12413. <https://doi.org/10.1111/desc.12413>

Kavanaugh, B. C., Parade, S., Seifer, R., McLaughlin, N. C. R., Tirrell, E., Festa, E. K., Oberman, L. M., Novick, A. M., Carpenter, L. L., & Tyrka, A. R. (2024). Childhood stress, gender, and cognitive control: Midline theta power. *Journal of Psychiatric Research*, *169*, 298–306. <https://doi.org/10.1016/j.jpsychires.2023.11.046>

Kokosi, T., Flouri, E., & Midouhas, E. (2021). The role of inflammation in the association between poverty and working memory in childhood. *Psychoneuroendocrinology*, *123*, 105040. <https://doi.org/10.1016/j.psyneuen.2020.105040>

Lamm, C., Troller-Renfree, S. V., Zeanah, C. H., Nelson, C. A., & Fox, N. A. (2018). Impact of early institutionalization on attention mechanisms underlying the inhibition of a planned action. *Neuropsychologia*, *117*, 339–346. <https://doi.org/10.1016/j.neuropsychologia.2018.06.008>

Lengua, L. J., Thompson, S. F., Ruberry, E. J., Kiff, C. J., Klein, M. R., Moran, L. R., & Zalewski, M. (2022). Concurrent and prospective effects of income, adversity, and parenting behaviors on middle‐childhood effortful control and adjustment. *Social Development*, *31*(3), 733–748. <https://doi.org/10.1111/sode.12562>

Lewis-Morrarty, E., Dozier, M., Bernard, K., Terracciano, S. M., & Moore, S. V. (2012). Cognitive Flexibility and Theory of Mind Outcomes Among Foster Children: Preschool Follow-Up Results of a Randomized Clinical Trial. *Journal of Adolescent Health*, *51*(2), S17–S22. <https://doi.org/10.1016/j.jadohealth.2012.05.005>

Li, M., Lindenmuth, M., Tarnai, K., Lee, J., King-Casas, B., Kim-Spoon, J., & Deater-Deckard, K. (2022). Development of cognitive control during adolescence: The integrative effects of family socioeconomic status and parenting behaviors. *Developmental Cognitive Neuroscience*, *57*, 101139. <https://doi.org/10.1016/j.dcn.2022.101139>

Lynch, K. S., & Widom, C. S. (2022). Childhood maltreatment and cognitive functioning in middle adulthood. *Child Abuse & Neglect*, *132*, 105791. <https://doi.org/10.1016/j.chiabu.2022.105791>

Maxfield, M., Li, X., & Widom, C. S. (2023). Childhood maltreatment and midlife cognitive functioning: A longitudinal study of the roles of social support and social isolation. *Neuropsychology*, *37*(8), 943–954. <https://doi.org/10.1037/neu0000911>

Motsan, S., Yirmiya, K., & Feldman, R. (2022). Chronic early trauma impairs emotion recognition and executive functions in youth; specifying biobehavioral precursors of risk and resilience. *Development and Psychopathology*, *34*(4), 1339–1352. <https://doi.org/10.1017/S0954579421000067>

Nikulina, V., & Widom, C. S. (2013). Child maltreatment and executive functioning in middle adulthood: A prospective examination. *Neuropsychology*, *27*(4), 417–427. <https://doi.org/10.1037/a0032811>

Nweze, T., Ezenwa, M., Ajaelu, C., Hanson, J. L., & Okoye, C. (2023). Cognitive variations following exposure to childhood adversity: Evidence from a pre-registered, longitudinal study. *eClinicalMedicine*, *56*, 101784. <https://doi.org/10.1016/j.eclinm.2022.101784>

Pears, K. C., Bruce, J., Fisher, P. A., & Kim, H. K. (2010). Indiscriminate Friendliness in Maltreated Foster Children. *Child Maltreatment*, *15*(1), 64–75. <https://doi.org/10.1177/1077559509337891>

Savopoulos, P., Brown, S., Anderson, P. J., Gartland, D., Bryant, C., & Giallo, R. (2022). Intimate partner violence during infancy and cognitive outcomes in middle childhood: Results from an Australian community‐based mother and child cohort study. *Child Development*, *93*(4). <https://doi.org/10.1111/cdev.13736>

Tibu, F., Sheridan, M. A., McLaughlin, K. A., Nelson, C. A., Fox, N. A., & Zeanah, C. H. (2016). Disruptions of working memory and inhibition mediate the association between exposure to institutionalization and symptoms of attention deficit hyperactivity disorder. *Psychological Medicine*, *46*(3), 529–541. <https://doi.org/10.1017/S0033291715002020>

Troller‐Renfree, S., Nelson, C. A., Zeanah, C. H., & Fox, N. A. (2016). Deficits in error monitoring are associated with externalizing but not internalizing behaviors among children with a history of institutionalization. *Journal of Child Psychology and Psychiatry*, *57*(10), 1145–1153. <https://doi.org/10.1111/jcpp.12604>

Westermann, N., Busching, R., Klein, A. M., & Warschburger, P. (2023). The Longitudinal Interplay between Adverse Peer Experiences and Self-Regulation Facets: A Prospective Analysis during Middle Childhood. *Research on Child and Adolescent Psychopathology*. <https://doi.org/10.1007/s10802-023-01117-1>

Yazgan, I., Hanson, J. L., Bates, J. E., Lansford, J. E., Pettit, G. S., & Dodge, K. A. (2021). Cumulative early childhood adversity and later antisocial behavior: The mediating role of passive avoidance. *Development and Psychopathology*, *33*(1), 340–350. <https://doi.org/10.1017/S0954579419001809>

**Supplementary Information 3**

*Characteristics of Studies Included in Meta-Analysis*

| **No** | **Author** | **Sample Characteristics** | **Original ACEs Type** | **ACEs Subtypes based on DMAP** | **CC Domain** | **CC Measurement Tools** | **Task Paradigm** |
| --- | --- | --- | --- | --- | --- | --- | --- |
| 1 | Almas et al. (2016) | Participants were recruited for a longitudinal study of foster care intervention for the Bucharest Early Intervention Project (BEIP) | Institutionalization | Deprivation | WM | WISC-IV Working Memory Index | WISC-IV Working Memory Index |
| 2 | Awada et al. (2023) | The study utilized data from the FFCWS, a longitudinal birth cohort study that followed 4898 families from their child's birth until the child was age 15 | Spanking  Community violence  Emotional abuse  Maternal intimate partner violence (IPV)  Physical abuse  Neglect  Lack of collective efficacy  Food insecurity  Maternal hardship  Poverty ratio baseline | Threat  Deprivation | WM | Digit span WISC-IV | Digit span |
| 3 | Bosquet Enlow et al. (2019) | Participants were mother-child dyads enrolled in the Programming of Intergenerational Stress Mechanisms (PRISM) study, a prospective pregnancy cohort. | Prolonged separation from caregiver  Serious medical incident  Experiencing a serious accident  Natural disaster  Sexual assault  Interpersonal violence | Threat & Deprivation | WM  IC | Nebraska Barnyard Task (NBT)  Go/No-go task - Fish-shark | NBT  Go/No-go |
| 4 | Brieant et al. (2022) | Data from the Adolescent Brain Study, an ongoing longitudinal study. | Socioeconomic status  Household chaos  Parent substance use  Parent depression  Negative life events | Threat & Deprivation | IC | Multi-Source Interference Task (MSIT) | MSIT |
| 5 | Clark et al. (2022) | Data from an eight-year longitudinal evaluation of an intervention for mothers and preschoolers who had been exposed to Intimate Partner Violence. | Intimate partner violence (IPV) | Threat | CF  IC | Trail Making Test A, B  Stroop Test - Color-word | TMT A  TMT B  Stroop |
| 6 | Colvert et al. (2008) | Samples were Romanian adoptees and within-UK adoptees. | Institutionalization | Deprivation | IC | Stroop Test - Color-word-trial2-trial1 | Stroop |
| 7 | Conradt et al. (2014) | Data were drawn from the Maternal Lifestyle Study, a multisite investigation of the effects of prenatal substance exposure in a longitudinal follow-up from 1 month to 16 years. | Physical threat  Sexual threat  Deprivation | Threat & Deprivation | WM | CANTAB Spatial working memory & Stocking of Cambridge | CANTAB SWM |
| 8 | Demers et al. (2022) | Participants were adults from a longitudinal sample first recruited when they were 6-12 through a research summer camp for low-income, high-risk children. | Physical threat  Sexual threat  Emotional threat  Deprivation | Threat & Deprivation | IC | Go/No-go task - International Affective Picture System (IAPS) negative background | Go/No-go Emo |
| 9 | Demeusy et al. (2018) | Data were drawn from a larger randomized clinical trial evaluating the efficacy of two preventive interventions for maltreated children. This study used a Department of Human Services (DHS), Child Protective Service (CPS) recruitment liaison to recruit infants with histories of maltreatment and their mothers. | Deprivation (lack of adequate supervision)  Moral-legal deprivation  Educational deprivation | Deprivation | WM | Three boxes stationary (spatial working memory)  Three boxes scrambled (non-spatial working memory) | Three boxes stationary  Three boxes scramble |
| 10 | Frenkel et al. (2020) | The sample was children taking part in a longitudinal study following child adoption into families after experiencing early institutional care. | Institutionalization | Deprivation | IC | Go/No-go task - The Zoo game | Go/No-go |
| 11 | Golm et al. (2021) | Samples are Romanian adoptees and UK never-institutionalized adoptees. | Institutionalization | Deprivation | IC | Go/No-go task | Go/No-go |
| 12 | Gustafsson et al. (2015) | Participants were a subsample of the Durham Child Health and Development Study (DCHDS), a longitudinal study of socioeconomically and racially diverse parents/caregivers living in and around a midsized southeastern city. | Intimate partner violence (IPV) | Threat | WM  CF  IC | Digit Span Backward  Flexible Item Selection Task (FIST)  Day-night task | Digit span  FSIT  Stroop |
| 13 | Harms et al. (2017) | Participants were a community sample of school-aged children and followed for a decade. | Foster care  Multiple placements  Parents die  Serious, chronic medical and mental health problems of parent/caregiver members  Long-term instability in parental employment  Severe interparental marital conflict  Parental separation  Extensive incarceration of one of the parents  Homeless  Several close parent/caregiver members die unexpectedly  Physical violent parents  Separation of the child from the parent/caregiver | Threat & Deprivation | IC | Go/No-go task | Go/No-go |
| 14 | Hunter et al. (2022) | Participants were in the National Longitudinal Study of Adolescent to Adult Health. | Stressful life events such as the loss of a loved one, legal troubles, and exposure to violence. | Threat & Deprivation | WM | Backward Digit Span | Digit span |
| 15 | Jankowski et al. (2017) | Participants were recruited from a larger, longitudinal randomized control trial of MTFC-P. | Institutionalization | Deprivation | IC | Go/No-go task | Go/No-go |
| 16 | Kavanaugh et al. (2023) | This current sample was part of a larger, longitudinal study of a sample of children (Ridout et al., 2019) | Composite of the number of SES stressors, contextual stressors, maltreatment types, and other traumatic events | Threat & Deprivation | IC | Go/No-go task | Go/No-go |
| 17 | Kokosi et al. (2021) | Participants were sampled from the Avon Longitudinal Study of Parents and Children (ALSPAC), an ongoing birth cohort study that recruited 14,541 pregnant women resident in Avon UK | Economic deprivation/hardship | Deprivation | WM | Counting span | Counting span |
| 18 | Lamm et al. (2018) | Participants were part of the Bucharest Early Intervention Project (BEIP), a randomized control trial comparing the effects of foster care as an alternative to institutional care for young children abandoned at birth and placed in institutions. | Institutionalization | Deprivation | IC | Go/No-go task | Go/No-go |
| 19 | Lengua et al. (2022) | Participants were mothers and children who were recruited from a university-hospital birth register, daycares/preschools, health clinics, and charitable agencies | Adolescent parent  Low education  Single parent  Residential instability  Parent/caregiver structure  Household density  Stress  Maternal depression. | Threat & Deprivation | IC | NEPSY Inhibitory Control subscale, Day-Night. Stroop Color Word Test, Bear-Dragon, Dimensional Change | Stroop |
| 20 | Lewis-Morrarty et al. (2012) | Participants were children with a history of foster care placement and were randomly assigned to the ABC intervention (Attachment and Biobehavioral Catch-up Intervention). | Institutionalization | Deprivation | CF | Dimensional Change Card Sort (DCCS) | DCCS |
| 21 | Li et al. (2022) | The current sample was generally representative of the region (Appalachian region of south-western Virginia) regarding household income and ethnicity. | Economy deprivation | Deprivation | IC | Multi-Source Interference Task (MSIT) | MSIT |
| 22 | Lynch et al. (2022) | Data were originally collected as part of a large prospective cohort design study that examined the long-term consequences of childhood maltreatment. | Physical Threat  Sexual Threat  Deprivation | Threat & Deprivation | CF  IC | Trail Making Test B  Stroop test | TMT B  Stroop |
| 23 | Maxfield et al. (2023) | Data used were collected as part of a large prospective cohort design study. | Extreme failure to provide adequate food, clothing, shelter, and medical attention to children  Physical abuse  Sexual abuse | Threat & Deprivation | CF | Trail Making Test A, B | TMT A  TMT B |
| 24 | Motsan et al. (2022) | Cohort of war-exposed preadolescents exposed to the same external trauma. | War | Threat | IC | CANTAB Stop signal task | CANTAB SWM |
| 25 | Nikulina et al. (2013) | Prospective study of court-substantiated maltreated children and controls followed into middle adulthood. | Deprivation (deficiencies in childcare)  Physical threat  Sexual threat | Threat & Deprivation | CF | Trail Making Test B | TMT B |
| 26 | Nweze et al. (2023) | Data came from the AVON Longitudinal Study of Parents and Children (ALSPAC), a multi-wave population cohort study that prospectively sampled. | Physical threat  Sexual threat  Inconsistent caregiving  Parent/caregiver instability  Caregivers threat  Maternal psychopathology  Maternal victimization  Parental legal problem  Neighborhood stress | Threat & Deprivation | WM  IC | N-Back Task  Stop Signal Task | N-Back  SST |
| 27 | Pears et al. (2010) | The sample used in the study was a subsample of children included in an efficacy trial to evaluate a treatment foster care program for preschool-aged children. | Physical threat  Sexual threat  Physical deprivation  Supervisory deprivation  Emotional maltreatment | Threat & Deprivation | IC | Modified Stroop task (night & day), Dimensional change card sort task, NEPSY attention/executive domain, Attention problems subscale of the child behaviour checklist | Stroop  DSSC  NEPSY |
| 28 | Savopuolus et al. (2022) | Data was drawn from a larger community-based longitudinal study of 1507 pregnant women. | Intimate partner violence (IPV) | Threat | WM  CF  IC | List Sorting Working Memory Test  Picture Sequence Memory Test  Dimensional Change Card Sort (DCCS)  Flanker Test | NIH-TB  NIH-TB  DCCS  Flanker |
| 29 | Tibu et al. (2016) | Participants were children from the Bucharest Early Intervention Project (BEIP), a longitudinal study of the effects of institutionalization. | Institutionalization | Deprivation | WM  CF  IC | CANTAB spatial working memory  Stocking of Cambridge (SOC)  Flanker Test | CANTAB SWM  SOC  Flanker |
| 30 | Troller-Renfree et al. (2016) | Participants were children from the Bucharest Early Intervention Project (BEIP), a longitudinal study of the effects of institutionalization. | Institutionalization | Deprivation | IC | Flanker Test | Flanker |
| 31 | Westermann et al. (2023) | Data were collected as part of a multifaceted prospective study investigating intrapersonal development risk factors in childhood and adolescence (PIER Study). | Adverse peer experiences | Threat | WM  CF  IC | Digit Span Backward WISC-IV  Dimensional Change Card Sort (DCCS)  Stroop Task | Digit span  DCCS  Stroop |
| 32 | Yazgan et al. (2021) | Participants were children followed in the Child Development Project, a longitudinal study of a community sample. | Sociocultural adversity  Parent/caregiving adversity  Peer adverse experience | Threat & Deprivation | IC | Go/No-go task | Go/No-go |

**Supplementary Information 4**

*Number of Studies and Effect Sizes Included in the Moderator Analysis for Working Memory*

|  | # of studies | *k* (# of effect sizes) |
| --- | --- | --- |
| **ACEs Subtypes**  Threat | 5 | 13 |
| Deprivation | 5 | 9 |
| Threat and Deprivation | 4 | 7 |
| **Task Paradigms** |  |  |
| Digit Span | 4 | 9 |
| CANTAB SWM | 2 | 3 |
| N-Back | 1 | 4 |
| Other | 5 | 13 |
| **Study Type** |  |  |
| Correlational | 8 | 19 |
| Comparative | 4 | 10 |

*Number of Studies and Effect Sizes Included in the Moderator Analysis for Cognitive Flexibility*

|  | # of studies | *k* (# of effect sizes) |
| --- | --- | --- |
| **ACEs Subtypes** |  |  |
| Threat | 4 | 10 |
| Deprivation | 2 | 3 |
| Threat and Deprivation | 3 | 4 |
| **Task Paradigms** |  |  |
| TMT B | 4 | 4 |
| FSIT | 1 | 3 |
| DCCS | 3 | 7 |
| Other | 3 | 3 |
| **Study Type** |  |  |
| Correlational | 4 | 10 |
| Comparative | 5 | 7 |

*Number of Studies and Effect Sizes Included in the Moderator Analysis for Inhibitory Control*

|  | # of studies | *k* (# of effect sizes) |
| --- | --- | --- |
| **ACEs Subtypes** |  |  |
| Threat | 5 | 10 |
| Deprivation | 9 | 44 |
| Threat and Deprivation | 10 | 24 |
| **Task Paradigms** |  |  |
| Go/No-go | 8 | 26 |
| Go/No-go Emo | 1 | 6 |
| MSIT | 2 | 14 |
| Stroop | 7 | 14 |
| Flanker | 3 | 13 |
| SST | 2 | 5 |
| **Study Type** |  |  |
| Correlational | 11 | 32 |
| Comparative | 12 | 46 |

**Supplementary Information 5**

*Newcastle-Ottawa Scale (NOS) Quality Assessment of the Included Articles*

| No | Article | Selection (/4) | Comparability (/2) | Outcome (/3) | Overall (/9) | Category (Poor/Fair/Good) |
| --- | --- | --- | --- | --- | --- | --- |
| 1 | Almas et al. (2016) | 2 | 1 | 3 | 6 | Fair |
| 2 | Awada et al. (2023) | 3 | 1 | 3 | 7 | Good |
| 3 | Bosquet Enlow et al. (2019) | 2 | 1 | 2 | 5 | Fair |
| 4 | Brieant et al. (2022) | 2 | 1 | 3 | 6 | Fair |
| 5 | Clark et al. (2022) | 2 | 1 | 3 | 6 | Fair |
| 6 | Colvert et al. (2008) | 2 | 1 | 3 | 6 | Fair |
| 7 | Conradt et al. (2014) | 2 | 1 | 3 | 6 | Fair |
| 8 | Demers et al. (2022) | 3 | 1 | 3 | 7 | Good |
| 9 | Demeusy et al. (2018) | 2 | 1 | 3 | 6 | Fair |
| 10 | Frenkel et al. (2020) | 2 | 1 | 3 | 6 | Fair |
| 11 | Golm et al. (2021) | 3 | 2 | 2 | 7 | Good |
| 12 | Gustafsson et al. (2015) | 2 | 2 | 2 | 6 | Fair |
| 13 | Harms et al. (2017) | 3 | 1 | 3 | 7 | Good |
| 14 | Hunter et al. (2022) | 1 | 0 | 2 | 3 | Poor |
| 15 | Jankowski et al. (2017) | 3 | 2 | 3 | 8 | Good |
| 16 | Kavanaugh et al. (2023) | 3 | 1 | 3 | 7 | Good |
| 17 | Kokosi et al. (2021) | 1 | 2 | 3 | 6 | Fair |
| 18 | Lamm et al. (2018) | 2 | 0 | 3 | 5 | Fair |
| 19 | Lengua et al. (2022) | 2 | 1 | 2 | 5 | Fair |
| 20 | Lewis-Morrarty et al. (2012) | 2 | 1 | 3 | 6 | Fair |
| 21 | Li et al. (2022) | 2 | 1 | 3 | 6 | Fair |
| 22 | Lynch et al. (2022) | 2 | 1 | 3 | 6 | Fair |
| 23 | Maxfield et al. (2023) | 3 | 1 | 3 | 7 | Good |
| 24 | Motsan et al. (2022) | 2 | 1 | 3 | 6 | Fair |
| 25 | Nikulina et al. (2013) | 2 | 1 | 3 | 6 | Fair |
| 26 | Nweze et al. (2023) | 3 | 1 | 3 | 7 | Good |
| 27 | Pears et al. (2010) | 2 | 1 | 3 | 6 | Fair |
| 28 | Savopuolus et al. (2022) | 2 | 1 | 3 | 6 | Fair |
| 29 | Tibu et al. (2016) | 2 | 1 | 3 | 6 | Fair |
| 30 | Troller-Renfree et al. (2016) | 2 | 0 | 3 | 5 | Fair |
| 31 | Westermann et al. (2023) | 3 | 1 | 3 | 7 | Good |
| 32 | Yazgan et al. (2021) | 3 | 1 | 3 | 7 | Good |
|  | **Mean** | 2.25 | 1.03 | 2.84 | 6.12 | Fair |

*Note.* Rating category: A study is categorized as poor if the selection domain scores 0 or 1, the comparability domain scores 0, or the outcome domain scores 0-1. A study is categorized as fair if the selection domain scores 2, the comparability domain scores 1 or 2, and the outcome domain scores 2 or 3 points. A study is categorized as good if the selection domain scores 3 or 4, the comparability domain scores 1 or 2, and the outcome domain scores 2 or 3. The maximum achievable quality score is 9.

**Supplementary Information 6**

*Age Distribution of Included Studies*


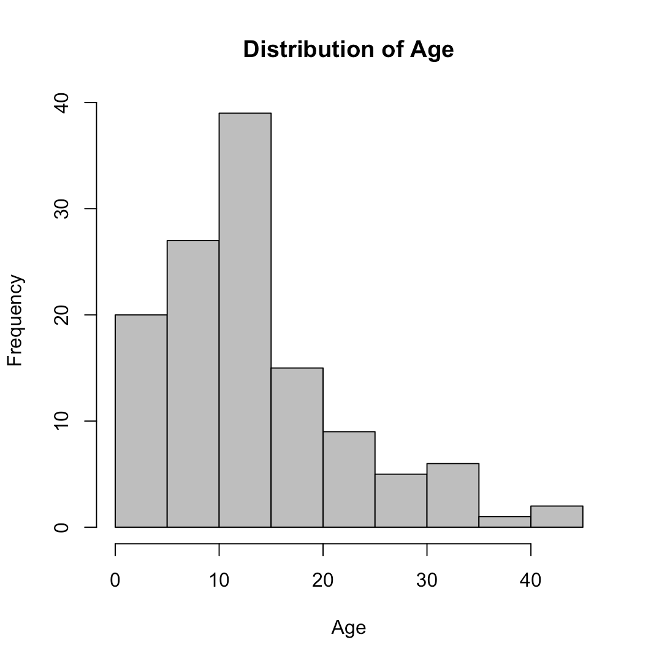

Supplement: sj-docx-1-tva-10.1177_15248380241286812 – Supplemental material for The Impact of Adverse Childhood Experiences on Cognitive Control Across the Lifespan: A Systematic Review and Meta-analysis of Prospective Studies [file sj-docx-1-tva-10.1177_15248380241286812.docx]
